# Deducing Phonon Scattering from Normal Mode Excitations

**DOI:** 10.1038/s41598-019-43306-3

**Published:** 2019-05-30

**Authors:** Anant Raj, Jacob Eapen

**Affiliations:** Department of Nuclear Engineering, North Carolina State University, Raleigh, NC 27695 USA

**Keywords:** Thermoelectric devices and materials, Atomistic models

## Abstract

While the quantum scattering theory has provided the theoretical underpinning for phonon interactions, the correspondence between the phonon modes and normal modes of vibrations has never been fully established; for example, the nature of energy exchange during elementary normal mode interactions remains largely unknown. In this work, by adopting a set of real asymmetric normal mode amplitudes, we first discriminate the normal and Umklapp processes directly from atomistic dynamics. We then demonstrate that the undulating harmonic and anharmonic potentials, which allow a number of interaction pathways, generate several total-energy-conserving forward and backward scattering events including those which are traditionally considered as quantum-forbidden. Although the normal mode energy is proportional to the square of the eigen-frequency, we deduce that the energy exchanged from one mode to another in each elementary interaction is proportional to the frequency – a quantum-like restriction. We anticipate that the current approach can be utilized profitably to discover unbiased scattering channels, many traditionally quantum forbidden, with complex anharmonicities. Our discovery will aid in the development of next-generation Peierls-Boltzmann transport simulations that access normal mode scattering pathways from finite temperature *ab initio* simulations.

## Introduction

Normal modes are non-interacting, non-decaying collective excitations of atomic vibrations – these are the eigen-states of the interacting harmonic Hamiltonian. In a crystal lattice with translational symmetry, the normal modes have eigen-frequencies that correspond to well-defined wave-vectors. Analogous to photons that are quantum excitations of an electromagnetic field, phonons are the quantum excitations of the lattice waves in a crystal^1–3^. Unlike photons, which arise as a particle-wave representation of an exact harmonic gauge potential, phonons are conceptualized from materials-specific anharmonic potentials, which allows a large diversity in their interactions, including among themselves^4^. Unsurprisingly, phonon interactions form the bedrock of our understanding of crystal response to external disturbances, particularly in the area of thermal transport – recent advances showcase unprecedented strides in phononics^5–10^, nanoscale and low-dimensional transport^11–20^, materials discovery^21–23^, and theoretical and simulation methods^24–34^.

Phonon-phonon interactions are usually treated as scattering events within the time-dependent perturbation theory. A key assumption in deriving the transition rates is that the anharmonic Hamiltonians add only small perturbations, and higher order contributions get progressively weaker. This assumption has been examined recently from two angles. Historically, the three-phonon interactions have been assumed to dominate energy transport with little or no contributions from higher-order processes. Recent work through the iterative solution of Peierls-Boltzmann transport equation (PBTE), however, has upended this notion by demonstrating appreciable contributions from the four-phonon scattering processes in both bulk and 2D materials^32,35^. Although the probability for elementary four-phonon excitations is lower than that of three-phonons, the less-restrictive conservation rules can accord a larger phase space for four and higher order phonon scattering interactions. Perturbative expansion is also critiqued for its inability to account for strong anharmonic interactions that are comparable to the harmonic interactions^36–38^. Such situations typically arise at temperatures comparable to the Debye temperature (Θ_*D*_), or near second order phase transitions^39^ and dynamic/displacive instabilities. Large amplitude atomic/ionic oscillations^40^ or off-center rattling^41,42^ can also break the translational invariance and the assumption of a weak perturbation.

Many-body atomistic dynamics^43,44^ with an arbitrarily accurate anharmonic Hamiltonian provides a direct approach for probing the interactions among the crystal normal modes. Loss of translation or lowering of symmetry associated with structural complexities can be accommodated, in principle, with atomistic dynamics to all orders of anharmonicity. Three serious limitations hamper the close correspondence between the phonons and normal modes. Phonons are bound by Bose-Einstein statistics^25,45^ while atoms conform to the Boltzmann statistics. Semi-classical quantum baths^46,47^ can potentially address this concern but their practicality is yet to be established fully^48^. Secondly, the traditional normal mode analysis (NMA) framework, which employs complex normal mode coordinates, does not distinguish the non-resistive normal (N) phonon interactions from the resistive Umklapp (U) phonon interactions^49^ – a serious drawback in identifying quantum-like phonon interactions, given the overarching importance of the U processes in thermal energy transport^1^. The final limitation on the correspondence between the normal modes and phonon modes is right at the heart of quantum mechanics in that the energy transfer between quantum objects is always proportional to the corresponding frequencies, but the nature of energy exchange during elementary normal mode interactions remains largely unknown.

In this work, we show  that the theoretical limitation of the traditional NMA analyses can be removed by adopting a set of real asymmetric normal mode amplitudes, which can distinguish lattice waves moving in opposite wave vector (i.e. in +**q** and −**q)** directions – a virtue that immediately endows the ability to discriminate N and U processes and the phase space associated with their interactions (see Methods section). Although the normal modes exchange energy continuously, they are localized in the reciprocal space and engage in discrete interactions. Crucially, we observe that the harmonic and anharmonic energies do not remain constant in time. The oscillating energies, which appear to be generic to all anharmonic systems, allow interaction pathways that have not been identified before including those which are traditionally considered as quantum-forbidden. Finally, using controlled cubic and quartic anharmonicity, we infer that the energy transferred from one normal mode to another in an elementary interaction is proportional to the corresponding frequencies – a surprising similarity to energy interchanges at the quantum level.

## Results

### Atomistic systems

We select linear Fermi-Pasta-Ulam (FPU)^50–52^ lattices with third and fourth order anharmonicities for illustrating the emergence of N and U modes. With the vibrations constrained to a single dimension, the lattices with cubic (FPU-α) and quartic (FPU-β) anharmonicities are specifically intended to probe the third and fourth order scattering processes, respectively. Historically, FPU lattices have been analyzed in the context of quasi-periodic or recurrence phenomena but in this work we have selected the parameters such that the lattices are ergodic and fully amenable to thermalization (see Methods section). The FPU models thus serve as surrogates of more sophisticated anharmonic systems. To illustrate the broader capability of the current approach, we also examine the modal interactions in graphene, which is modeled through an optimized Tersoff potential^53^ that includes anharmonicities of all orders. While the phonon dispersion is reasonably captured by the optimized Tersoff potential, the higher order anharmonicities of this potential may not be accurate. The choice of the models reflects our central purpose of demonstrating the scattering of normal modes and evaluating the extent to which it concurs with or diverges from the established quantum phonon scattering theory.

### Conservation of crystal momentum – N and U modes

**Figure 1:**
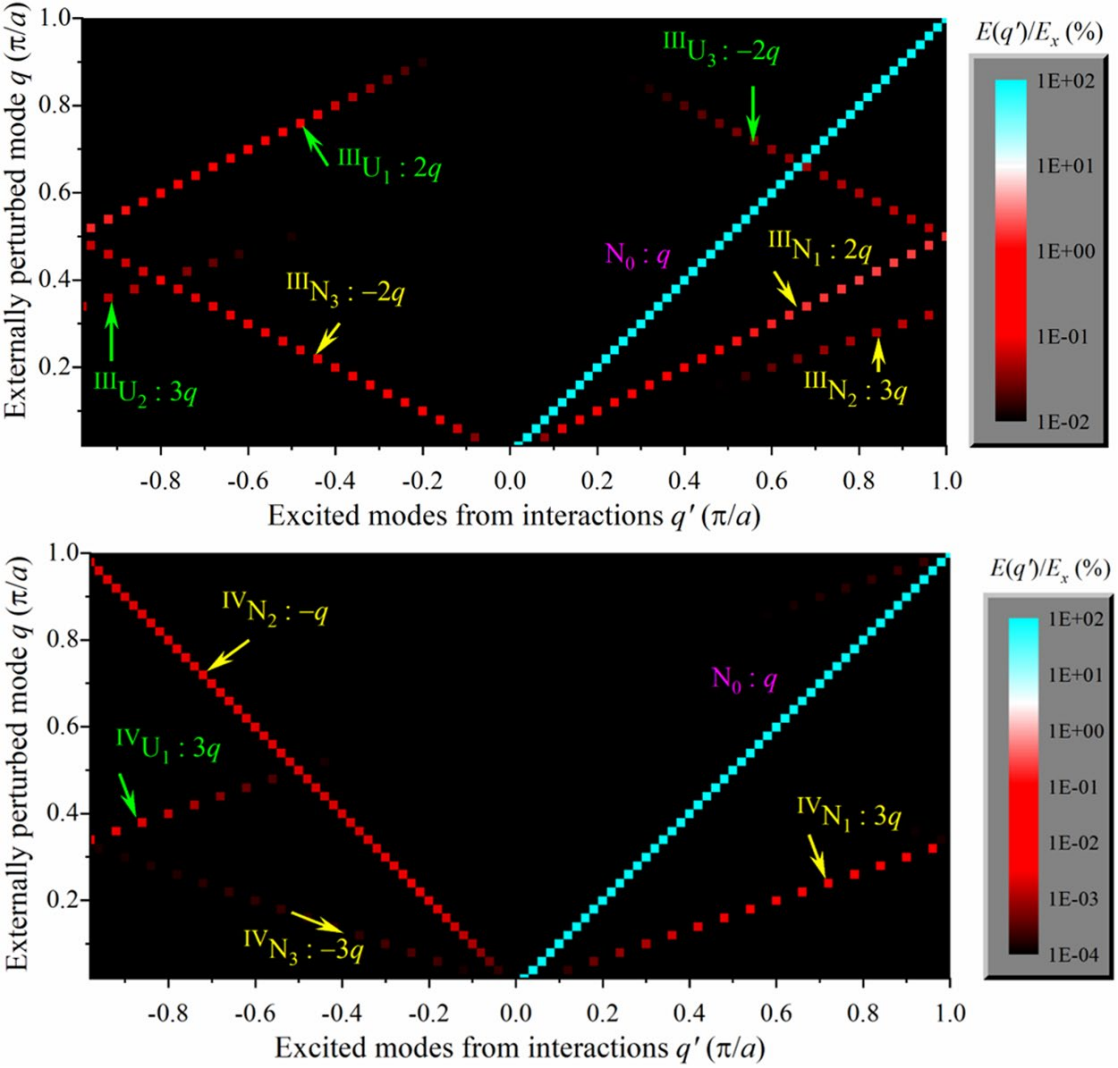
Normal mode excitations (*q*′, *x*-axis) from externally perturbed modes (*q*, *y*-axis) on a FPU lattice with only cubic anharmonicity (FPU-α, top) and only quartic anharmonicity (FPU-β, bottom); the scale on the right denotes the energy of the corresponding modes. With all the atoms initially at their equilibrium positions and without any initial kinetic energy, a single mode *q* is first perturbed with an excess energy *E*_*x*_. This external perturbation engenders additional interactions or excitations. The perturbation protocol is then repeated sequentually for several values of wavevectors (see Methods section). The excitations comprise of both N (yellow) and U (green) processes associated with the elementary merging overtones: *q* + *q* → *2q* + *g*; *q* + 2*q* → *3q* + *g* for the FPU-α lattice with only cubic (III) anharmonicity, and *q* + *q* + *q* → *3q* + *g* for the FPU-β lattice with only quartic (IV) anharmonicity. The ability to capture the N and U modes separately arises through the use of real asymmetric normal mode amplitudes. Although not shown, splitting modes emerge at later times. Modes with negative wavevectors that do not fall in the merging or splitting categories also appear (−*q*, −2*q*, −3*q*). The numerical protocol for generating the excited modes is detailed in Supplemental Information A.

In Fig. 1 we delineate the excited normal modes (*x*-axis) following perturbation of a single mode (*y*-axis) in a FPU-α lattice (top) and a FPU-β lattice (bottom) – the details are given in the Methods section and in Supplemental Information A. With all the atoms initially at their equilibrium positions without any kinetic energy, a single mode is perturbed first, and the resultant excited normal modes, which are measured by the magnitude of the modal energies, are tracked during a short observation time window of 0.4 units. As expected, the most prominent excited mode is N_0_, which corresponds to the perturbing wavevector *q*. The next prominent excitations are the overtones of the primary modes: *q* + *q* → *2q*; *q* + 2*q* → *3q* for the FPU-α lattice (with only cubic anharmonicity) and *q* + *q* + *q* → *3q* for the FPU-β lattice (with only quartic anharmonicity); these excitations are analogous to the three and four phonon merging events, respectively. More strikingly, the corresponding U-type processes (*2q* + *g* and *3q* + *g*), also emerge. Thus by faithfully capturing the expected N and U processes, which is made possible by the asymmetric normal mode coordinates, we provide the first key correspondence between the normal mode interactions and the phonon modes. Since only a single mode is perturbed initially, merging events are dominant in the short time of observation (0.4 units). At longer times, or with more complex perturbations involving multiple modes, both Class I merging and Class II splitting events^4^ can be identified (results not shown). A curiously peculiar set of excitations involves the negative wavevectors, which does not fall in the aforesaid classification. The −2*q* mode for the FPU-α lattice, and the −*q* and −3*q* modes for the FPU-β lattice conserve crystal momentum and they are similar to the overtone modes previously described, however, with a negative sign. Similar overtones and Umklapp reflection at the zone boundary are observed in graphene, which is governed by a fully anharmonic interatomic potential (Supplemental Information B). Interestingly, negative modes appear prominently in graphene too as shown in Supplemental Information C and D.

### Time-varying harmonic and anharmonic energies

**Figure 2:**
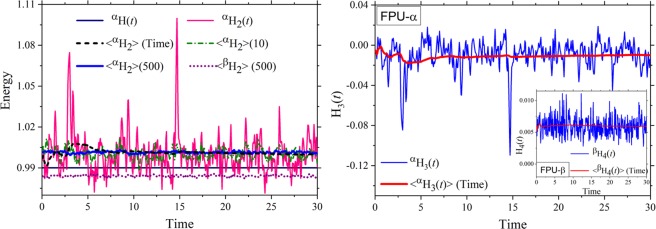
(Left) Variation of harmonic energy (H_2_) and total energy (H) in time for the FPU-α/β systems at a temperature of 0.01. Although the total energy remains a constant in time, the cubic (α) and quartic (β) harmonic Hamiltonians show significant fluctuations. Only with increasing number of time or phase-space averages (the number in the parenthesis depicts the number of averages from different initial conditions), the harmonic energy approaches a constant value. The right panel shows the temporal variation of the instantaneous anharmonic energy ^α^H_3_(*t*) = ^α^H(*t*) − ^α^H_2_(*t*) from a single phase space trajectory along with the (running) time-averaged ^α^H_3_ for the FPU-α lattice. The corresponding anharmonic energy ^β^H_4_(*t*) = ^β^H(*t*) − ^β^H_2_(*t*) and the (running) time average for the FPU-β lattice are shown in the inset. Note that ^α^H_4_(*t*) and ^β^H_3_(*t*) are both zero, by construct.

To understand the origin of the excited negative modes, we first examine the temporal behavior of the harmonic energies in Fig. 2 for ***well-equilibrated*** FPU lattices. We simulate these systems with small kinetic energies such that they are nearly harmonic. Without using an external thermal bath or rescaling, both FPU systems are allowed to attain a steady temperature *T* of 0.01, which is sufficiently close to 0 *K* and almost two orders smaller than Θ_D_, the Debye temperature. The left panel in Fig. 2 depicts the temporal behavior of the instantaneous harmonic and total energy from a single run as well as the harmonic energy averaged over time and different initial phases. The right panel shows the instantaneous and average behavior of the anharmonic energy (H_3_/H_4_) for the FPU-α/β systems.

Most notably, the harmonic energy (H_2_) is not constant in time even at low temperatures. Although H_2_ approaches a steady value when averaged over time or a large number of initial phases, the fluctuations are conspicuously large for a single run as shown in Fig. 2 (left panel). Since the normal modes interact in any given phase trajectory, the oscillating harmonic energy deserves more attention because the theory of quantum phonon interactions, which is largely formulated through the time-dependent perturbation theory or through renormalization^36^, assumes a constant harmonic energy for all singular events such as three-phonon or four-phonon interactions. Further, the standard quantum phonon theory prescribes constant anharmonic energies as well without allowing any exchange between the harmonic and anharmonic Hamiltonians. Normal mode energies in atomistic systems, contrarily, portray a time varying behavior that is distinctly different. For any given initial phase, H_2_ serves as an energy bank from which the anharmonic Hamiltonians can borrow or return small amounts of energy. The instantaneous fluctuations of the anharmonic energies H_3_ and H_4_ (right panel of Fig. 2) reflect this energy transfer between H_2_ and H_3_, and H_2_ and H_4_, respectively. Expectedly, with time or phase space averaging, the energy fluctuations become small. Since ^β^H_4_ is always positive, ^β^H_2_ is lower than the total energy for FPU-β system. Although ^α^H_3_ can take positive or negative values, ^α^H_3_ for the FPU-α system that is investigated here is negative. We will return to the limiting constancy of harmonic and anharmonic energies on phase-space/time averaging in the context of energy conservation in phonon-phonon interactions within the quantum framework.

The oscillatory nature of energies is not unique to the particular systems that are investigated here; rather it appears to be universal to all vibratory anharmonic atomistic systems as there are no inherent physical mechanisms to maintain harmonic and anharmonic energies that are invariant in time. The undulatory nature of the Hamiltonians, however, confers additional freedom for the normal modes to interact in ways not possible with constant Hamiltonians. Since the total energy is constant, we infer that portions of harmonic energy are continuously interchanged with the anharmonic components.

### Negative normal modes

The origin of the negative modes is rooted in the time variation of the harmonic/anharmonic energies, and is best illustrated by examining the theoretical structure of the Hamiltonians with the real asymmetric normal mode amplitudes in three dimensions. For simplicity, we focus only on the cubic term (the full derivation is given in Supplemental Information E), which is given by:

1 $${H}_{3}(t)=\sum _{{\bf{q}},p}[\begin{array}{c}(L\times A({{\bf{q}}}_{1},t)A({{\bf{q}}}_{2},t)A({{\bf{q}}}_{3},t)\cos ((w({{\bf{q}}}_{1})+w({{\bf{q}}}_{2})+w({{\bf{q}}}_{3}))t+{\varphi }_{1}))+\\ (L\times A({{\bf{q}}}_{1},t)A({{\bf{q}}}_{2},t)A(-{{\bf{q}}}_{3},t)\cos ((w({{\bf{q}}}_{1})+w({{\bf{q}}}_{2})-w({{\bf{q}}}_{3}))t+{\varphi }_{2}))+\\ (L\times A({{\bf{q}}}_{1},t)A(-{{\bf{q}}}_{2},t)A({{\bf{q}}}_{3},t)\cos ((w({{\bf{q}}}_{1})-w({{\bf{q}}}_{2})+w({{\bf{q}}}_{3}))t+{\varphi }_{3}))+\\ (L\times A({{\bf{q}}}_{1},t)A(-{{\bf{q}}}_{2},t)A(-{{\bf{q}}}_{3},t)\cos ((w({{\bf{q}}}_{1})-w({{\bf{q}}}_{2})-w({{\bf{q}}}_{3}))t+{\varphi }_{4}))\end{array}]$$

The above time-dependent anharmonic Hamiltonian *H*_3_(*t*) is summed over all polarizations *p* (not shown explicitly above) and wavevectors that satisfy **q**_1_ + **q**_2_ + **q**_3_ = **g** (for the FPU lattices, the above expression reduces to a single dimension). Equation (1) is completely analogous to the anharmonic expression involving the creation and annihilation operators^3,4^. The first term allows simultaneous creation and annihilation of three normal modes while the remaining three cover all possible combinations for the merging and splitting processes. Interestingly, the two spontaneous processes, which correspond to the first term, are deemed impossible quantum mechanically as they violate the H_3_ energy constancy (for specific three-phonon interactions) but this restriction is true only if H_3_ is taken as a constant. For anharmonic atomistic systems, the Hamiltonians H_2_ and H_3_ are time varying as discussed previously, and thus both simultaneous creation and destruction events can occur without violating total energy (H) conservation. For example, if we consider the −2*q* mode in the FPU-α system (see Fig. 1) – this mode does not correspond to either a merging or a splitting mode, but it can arise through a simultaneous creation N process: 0 → *q* + *q* + (−2*q*). In the traditional viewpoint, each of the partitions of the total Hamiltonian (H_2_, H_3_, H_4_ …) is assumed to be constant; with such a restriction, energy is conserved for each partition of the Hamiltonian. The simulations on FPU chains (and graphene), however, show that the partitions of total Hamiltonian are not constant, but they fluctuate with time. Thus the anharmonic Hamiltonians (H_3_, H_4_ …) can borrow or return small amounts of energy to harmonic energy (H_2_) bank as mentioned previously. This energy exchange allows the process: 0 → *q* + *q* + (−2*q*), which is traditionally considered as a simultaneous creation process. We emphasize here that simultaneous creation or annihilation processes do not violate the energy conservation law since at all times the total Hamiltonian (H) remains constant. The corresponding U process: 0 → *q* + *q* + (−2*q*) + *g* is also discernible and its magnitude is comparable to the N process. Very feeble −*q* modes, which do not appear on the chosen scale in Fig. 1, also emerge in the FPU-α lattice. They can either be formed through a splitting process *q* → *2q* + (−*q*) or through a decay process −2*q* → (−*q*) + (−*q*). Thus the number of possible normal mode interactions is greatly enhanced by the time-varying Hamiltonians.

For the FPU-β lattice, the −*q* mode, which is quite notable occurs through the decay process *q* → *q* + *q* + (−*q*). Thus the backward scattering normal mode −*q* appears prominent largely from the simplicity of the combination rule for a quartic Hamiltonian. The less conspicuous −3*q* mode, similar to the −2*q* mode in the FPU-α lattice, can arise from a spontaneous creation event: 0 → *q* + *q* + *q* + (−3*q*). The magnitude of the corresponding U processes for the negative modes with the FPU-β chain is insignificant for the short window of observation. With due passage of time, secondary events emerge leading to a plethora of normal mode interactions. Interestingly, negative −**q**_*LA*_ (longitudinal acoustic) and −**q**_*LO*_ (longitudinal optic) modes emerge in graphene following an excitation of a longitudinal LA mode (see Supplemental Information C and D). These modes ensue from four-phonon creation events: ${{\bf{q}}}_{LA}\to {{\bf{q}}}_{LA}+{{\bf{q}}}_{LA}+(-{{\bf{q}}}_{LA})$ and ${{\bf{q}}}_{LA}\to {{\bf{q}}}_{LA}+{{\bf{q}}}_{LA}+(-{{\bf{q}}}_{LO})$, which are remarkably identical to those observed with the FPU-β lattice that interacts only through a quartic potential. Such simultaneous creation (and annihilation) events are facilitated only through the time varying harmonic and anharmonic Hamiltonians, and appear to be generic and not specific to a particular kind of interatomic potential or dimensionality.

### Frequency of the excited energy oscillations

**Figure 3:**
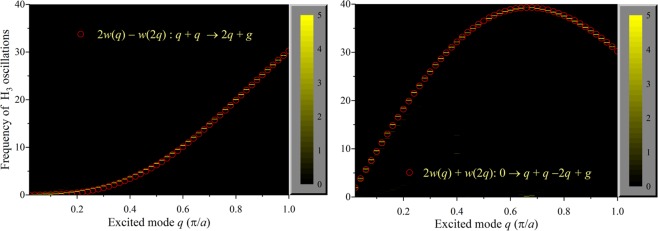
Comparison between the theoretical and computed frequencies of the modal energy oscillations in a FPU-α lattice. The left panel corresponds to the energy of mode 2*q* due to the merging interaction: *q* + *q* → 2*q* + *g* and the right panel corresponds to the energy of mode −2*q* from the simultaneous creation of three normal modes: 0 → *q* + *q* + (*−*2*q*) + *g*. The expected theoretical frequencies are depicted by the open circles while the computed frequencies are delineated by the short horizontal line segments, which are mostly coincident with the open circles. The color scale corresponds to the magnitude of the normalized Fourier transform of the deviation of the energy associated with the 2*q* mode (left) and the −2*q* mode (right).

The temporal behavior of the anharmonic Hamiltonian is regarded to have only a weak dependence on the rate of change of the amplitude *A*(**q**, *t*). Inspection of Eq. (1) reveals that the rate of energy that is exchanged in a normal mode interaction will then be proportional to the corresponding cosine term. For example, the energy corresponding to the most prominent 2*q* mode in the FPU-α lattice that is formed by the merging event: *q* + *q* → *2q* + *g*, is expected to oscillate with a distinctive frequency: 2*w*(*q*) − *w*(2*q*). Similarly, the expected frequency of the energy oscillations for the −2*q* mode will be: 2*w*(*q*) + *w*(2*q*). It is possible, therefore, to estimate the anticipated frequencies of all the modes that can be rationalized from crystal momentum conservation. Numerically, the excited frequencies of the energy exchange rate can be computed by evaluating the normalized Fourier transform of the deviation of the energy associated with each mode involved in any given interaction. In Fig. 3, we compare the numerical and the theoretical values of energy oscillations for the *2q* mode (left) and the −2*q* mode (right). Impressive agreement is observed between the theoretical predictions and the results from the simulations for the particular frequency set: 2*w*(*q*) ± *w*(*q*), which corresponds to ±2*q* modes. Although not shown, we have verified that the conformity is excellent for the other pathways in the FPU systems and graphene. Thus we can identify the frequencies of the energy oscillations of the normal modes that participate in different anharmonic interactions using simulations, as well as predict them with reasonable fidelity from the structure of the anharmonic Hamiltonian that is cast using the real asymmetric normal mode amplitudes. While the conservation of crystal momentum can allow several possibilities, the frequency analysis of the anharmonic energy oscillations can assist in isolating the pertinent normal mode interactions.

### Quantum-like energy exchange among the normal mode interactions

The structure of the anharmonic Hamiltonians also embodies information on energy exchange between different modes. With Eq. (1) as reference, it is evident that for H_3_(*t*) to remain finite on an average, the arguments of the cosine functions should tend to become zero. The simulations demonstrate that the mean value of H_3_(*t*) is finite and nearly a constant when averaged over a certain time period or different initial phases (see Fig. 2); in this regard, the simulated FPU systems are ergodic with the chosen parameters across the period of observation. With vanishing cosine arguments we observe the emergence of quantum-like frequency conditions: *w*_1_ ± *w*_2_ ± *w*_3_ = 0. Interestingly, this so-called resonant condition cannot be satisfied for finite wave-vectors for a FPU-α lattice along a particular phase space trajectory. However, the normal mode interactions, when averaged over time (or phase space), can mimic the distinctive energy conservation rule for quantum phonon interactions.

**Figure 4:**
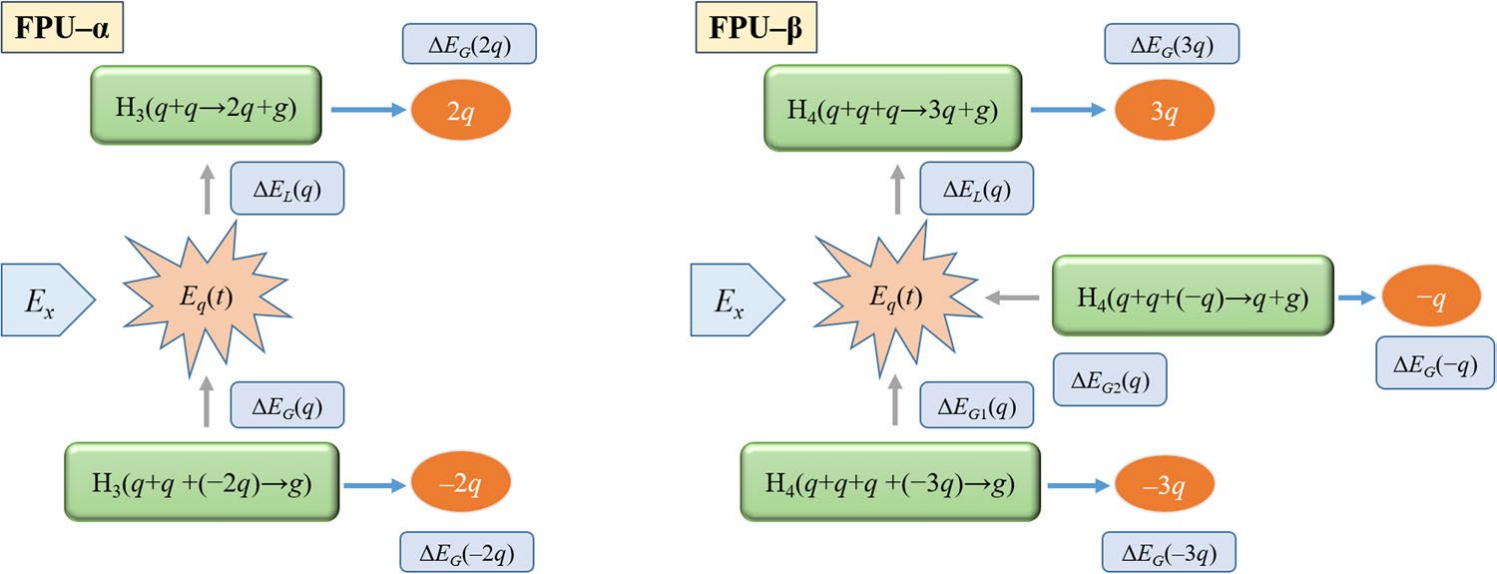
Pictorial representation of the energy transfer for the dominant ±2*q* modes in the FPU-α lattice (left), and ±*q* and ±3*q* modes in the FPU-β lattice (right), immediately following a single mode (*q*) perturbation.

One final question that remains is on the energy interchange between the discrete normal mode interactions in any given phase space trajectory. Taking advantage of the symmetry but not the equivalence between ±*nq* modes, we now proceed to make a quantitative comparison of the ratio of energies exchanged in specific sets of normal mode interactions. We now go back to the protocol that has generated the modes shown in Fig. 1 where each mode *q* is independently perturbed by excess energy *E*_*x*_. The externally perturbed mode *q* can hold on to this energy *E*_*q*_ only momentarily as plane wave modes cannot be sustained indefinitely in an anharmonic lattice. When the crystal momentum conditions are satisfied, the modes interact through the time-dependent anharmonic Hamiltonian that takes finite values. Since the normal modes exchange energy continuously, only a global energy balance can be performed; we therefore choose the most dominant modes following the excitation. The energy transfer for the dominant ±2*q* modes in the FPU-α lattice, and ±*q* and ±3*q* modes in the FPU-β lattice is depicted pictorially in Fig. 4. For the FPU-α lattice, the energy conservation is stated as: *E*_*q*_ = *E*_*x*_ + Δ*E*_*G*_(*q*) − Δ*E*_*L*_(*q*), where Δ*E*_*G*_(*q*) and Δ*E*_*L*_(*q*) are the energy gained by mode *q* in creating the −2*q* mode, and the energy lost by the mode *q* in establishing a 2*q* mode, respectively. The energies of ±*nq* modes are not the same with the real asymmetric normal mode amplitudes but the symmetry of the dispersion relationship grants the condition: *λ* = Δ*E*_*G*_(2*q*)/Δ*E*_*L*_(*q*) = Δ*E*_*G*_(−2*q*)/Δ*E*_*G*_(*q*). Note that we have only assumed that the energy exchanged by a particular normal mode is a unique function of its frequency. We thus can express *λ*, which is the ratio of energy exchanged during the modal interactions as: *λ*^−1^ = (*E*_*q*_ − *E*_*x*_)/(Δ*E*_*G*_(−2*q*) − Δ*E*_*G*_(2*q*)). The question that we are seeking now is whether this ratio is proportional to the ratio of the corresponding frequencies as prescribed by the quantum theory.

**Figure 5:**
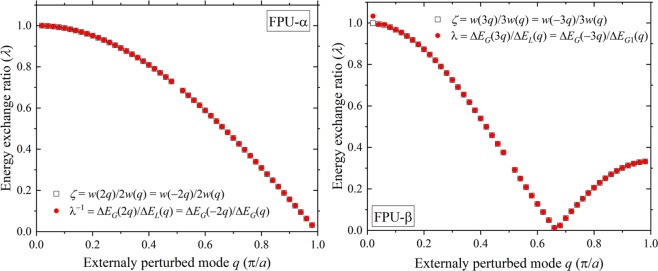
(Left) Ratio (λ) of the energy gained by mode 2*q* in the Class I merging process: *q* + *q* → 2*q* + *g* to that lost by mode *q*, or of the energy gained by mode −2*q* in the simultaneous creation event: 0 → *q* + *q* + (−2*q*) + *g* to that gained by mode *q*, in a FPU-α lattice. The quantum-like expected ratio (*ζ*) is depicted by the open squares. (Right) Corresponding ratios for the FPU-β lattice with the dominant processes *q* + *q* + *q* → 3*q* + *g*, 0 → *q* + *q* + *q* + (−3*q*) and *q* → *q* + *q* + (−*q*). The agreement between the energy ratio and the expected frequency ratio *ζ* shows a surprising quantum-like energy exchange between the normal modes.

In Fig. 5 (left), we plot *λ* for different excited modes along with the expected frequency ratio *ζ* = *w*(2*q*)/2*w*(*q*) = *w*(−2*q*)/2*w*(*q*) that is accessible from the phonon dispersion relationship. We can observe an impressive agreement between the ratios *λ* and *ζ*. We further test the proportionality of energy interchange in a FPU-β lattice, for which ±3*q* and ±*q* modes are most prominent (see Figs 1 and 4). The energy balance now has to account for both the interactions that have similar energies and can be written as: *E*_*q*_ = *E*_*x*_ + Δ*E*_*G*__1_(*q*) + Δ*E*_*G*__2_(*q*) − Δ*E*_*L*_(*q*), where Δ*E*_*G*__1_(*q*) and Δ*E*_*G*__2_(*q*) are the energies gained by mode *q* in forging the −3*q* mode and −*q* mode, respectively, and Δ*E*_*L*_(*q*) is the energy lost by mode *q* in establishing a 3*q* mode. As before, by letting *λ* = Δ*E*_*G*_(3*q*)/Δ*E*_*L*_(*q*) = Δ*E*_*G*_(−3*q*)/Δ*E*_*G*__1_(*q*), and Δ*E*_*G*__2_(*q*)/Δ*E*_*G*_(−*q*) = 1, we arrive at: *λ*^−1^ = (*E*_*q*_ − *E*_*x*_ − Δ*E*_*G*_(−*q*))/(Δ*E*_*G*_(−3*q*)  − Δ*E*_*G*_(3*q*)). In Fig. 5 (right panel), we compare the value of *λ* with *ζ* = *w*(3*q*)/3*w*(*q*) = *w*(−3*q*)/3*w*(*q*); Both ratios are accessible, *λ* from the simulations and *ζ* from the phonon dispersion relationship. The ratios again show a near-perfect agreement, which highlights the quantum-like proportionality of energy exchange between the normal modes for the FPU-β lattice. If the observation window is extended, more interactions would take place and a similar analysis would need a careful consideration of all the dominant processes. The remarkable agreement between the two ratios *λ* and *ζ* for all excited modes (*q*) reveals a rather curious nature of normal mode interactions. *Although the energy of the normal modes is proportional to the square of the eigen frequencies*, *the energy exchanged during discrete interaction events is proportional to the corresponding frequencies*. Thus the ratio of energy interchange among the participating normal modes appears in the same proportion as that stipulated by quantum rules.

## Discussion

While phonons are doubtless quantum objects, their roots are embedded in the vibrational normal modes that exchange energy continuously. A fundamental question naturally arises: under what conditions can the normal modes be treated as perfect quantum entities? In the quantum scattering theory, energy is not continuously exchanged and the discrete events that materialize from interactions are not necessarily well-defined. Several approximations are made in the scattering theory – the main one being the existence of well-defined quantum states before and after scattering. Therefore, the scattering theory presumes a well-defined set of plane waves changing sharply to another following an idealized scattering event, conserving both energy and momentum^3^. The theory, however, does not stipulate the nature of the event or the time duration for such an event to take place; thus multiple virtual states that can violate energy conservation are naturally accommodated within a composite scattering event that includes several multi-phonon processes. Although the elementary phonon interactions are usually limited to first order in the perturbing Hamiltonian, higher order perturbation expansions can lead to multi-phonon processes. For example, an overall or composite four-phonon interaction can take place by combining two three-phonon interactions *via* an intermediate state^3^. While energy is conserved for the composite event, it is not, in general, for the virtual or intermediate states.

As highlighted in this work, the Hamiltonians (H_2_, H_3_, H_4_, *etc*.) of an anharmonic vibratory system, even at low thermal energies, are time-dependent by nature, and can generate a large number of pathways for normal modes to interact. A straightforward incorporation of the time-dependent Hamiltonians, say, from high-fidelity *ab initio* atomistic dynamics simulations at finite temperatures, into the quantum phonon scattering theory can provide a direct correspondence to the normal mode interactions. However, by taking advantage of the near-constancy of the Hamiltonians (H_2_, H_3_, *etc*.) over time or with different phase-space averaging – both are equivalent for ergodic systems – an alternate approach can be construed that is identical to the current method of using time-independent Hamiltonians. The interpretation of a phonon scattering event then becomes different. Instead of an elementary phonon event that is characterized by a first order process, say, a three phonon merging/decaying process that can be described with an exact time-independent Hamiltonian H_3_, the composite phonon interaction with an ensemble-averaged constant Hamiltonian <H_3_> will now include ***all possible*** elementary phonon events. It can also include those which are traditionally considered impossible such as the simultaneous creation or annihilation of normal modes as intermediate processes, whose presence is significant as shown by the simulations of FPU lattices and graphene in this work. Because a composite event corresponds to a constant Hamiltonian, energy conservation can be formalized using the standard Dirac delta function for the overall process. Interestingly, a recent work recommends the use of a heavy-tailed continuous Lorentz distribution (for regularizing the Dirac delta function), which physically corresponds to energy conservation encompassing all three-phonon interactions^54^.

### Concluding remarks

We have demonstrated in this work that the normal modes, which can be extracted in atomistic systems using real asymmetric normal mode amplitudes, have the ability to discern both normal and Umklapp processes. Although the normal modes exchange energy continuously, they are localized in the reciprocal (**q**, *w*) space and engage in discrete interactions that comply with the conservation of crystal momentum. The mathematical structure of the time-varying third order anharmonic Hamiltonian reveals that the normal modes, on an average, exchange energy with the quantum-like frequency condition *w*_1_ ± *w*_2_ ± *w*_3_ = 0. More remarkably, we observe that the energy interchanged in a particular normal mode interaction is proportional to the corresponding frequencies for both cubic and quartic anharmonicities – a singularly important property that establishes a close correspondence to phonons. The normal mode interactions, however, differ from the phonon modes on the usual interpretation of energy conservation – the similarities and differences between them are listed in Table 1.

**Table 1:** Characteristics of normal mode and phonon mode interactions.

	Normal Modes	Phonon Modes
Energy exchange	Continuous.	Discrete.
Scattering events	Local in (**q**, *w*) space. Theoretically described through real asymmetric normal mode amplitudes that can resolve both N and U processes.	Local in (**q**, *w*) space. Theoretically described through the second quantization operators.
Crystal momentum	Conserved.	Conserved.
Interaction Hamiltonians (H_2_, H_3_, H_4_…)	Inherently time-dependent for all anharmonic lattices at finite temperatures; H(*t*) is the only instantaneous constant of motion. Each Hamiltonian approaches constancy with ensemble or time averaging.	Time-invariant harmonic and anharmonic Hamiltonians (H_2_, H_3_, H_4_…) are generally assumed. Within the scattering theory, perturbative or non-perturbative, this condition can be relaxed.
Simultaneous creation and annihilation processes	Emerge naturally by borrowing or forfeiting energies from/to H_2_. Responsible for negative backscattering modes, which provide additional scattering pathways.	Theoretically allowed but discarded on the basis of strict energy conservation for each operating Hamiltonian.
Energy conservation and frequency relationship with cubic anharmonicity^§^	Theoretically, a finite H_3_ corresponds to the frequency condition: *w*_1_ ± *w*_2_ ± *w*_3_ = 0, on an average. For discrete events, normal modes exchange energy proportional to their frequencies^●^ similar to phonon modes.	Energy conservation is enforced, separately, for merging/splitting scattering events, which leads to the condition *w*_1_ ± *w*_2_ ± *w*_3_ = 0. Intermediate processes that can violate energy conservation are allowed.
Statistics	Average properties are governed by the Boltzmann statistics, and thus strictly applicable above the Debye temperature (Θ_D_). Bose-Einstein statistics can be theoretically incorporated with quantum baths or through path integral molecular dynamics.	Phonon are governed by Bose-Einstein statistics which collapse to Boltzmann statistics at high temperatures.
Attributes	Interactions emerge naturally in atomistic systems governed by any order of anharmonicity. No foreknowledge is needed on the order of interactions or scattering avenues. Finite temperatures can be accommodated easily. Accuracy of the interactions largely depends on the fidelity of the anharmonic interatomic forces; individual Hamiltonians are not needed except when required for further analysis. To better understand the processes, more work is needed on complex systems.	Anharmonic force constants are needed to enumerate the phonon interactions. Prior knowledge on the order of interaction is typically needed and there are no general theoretical ways to estimate the highest operating anharmonicity or the associated scattering routes that proliferate with increasing anharmonicity. Usually limited to temperatures lower than Θ_D_; renormalization allows theoretical extension to higher temperatures. Higher order anharmonicties (above H_3_) typically incur heavy computational expense.

^§^Analogous contributions arise for higher order anharmonic terms. ^●^Demonstrated numerically for both cubic and quartic interactions in a FPU lattice.

The time dependent Hamiltonians (H_2_, H_3_, H_4_…) – an intrinsic property of all anharmonic lattices, even at low temperatures, can allow a multitude of elementary normal mode interactions that may not conserve energy. We anticipate that such additional scattering channels, yet to be discovered with more complex Hamiltonians through the current approach, can be treated theoretically without further difficulty within the quantum phonon scattering theory. The numerical experiments on FPU-α/β lattices (and graphene) provide weighty evidence for considering normal modes as analogues of quantum phonon modes, and they open up a new mode of theoretical and numerical inquiry into phonon interactions.

In the framework of Peierls-Boltzmann equation (PBTE), the thermal conductivity of a solid dielectric material is governed by the phonon scattering channels, particularly by the Umklapp processes that determine the thermal dissipation in a material. While normal processes are non-resistive, they can create additional pathways for the Umklapp processes. The current work shows that atomistic simulations can probe and enumerate the scattering pathways, which emerge naturally without any prior assumption on the order or the type of scattering. In the case study with FPU lattices and graphene (see Supplemental Information), our method identifies the commonly known scattering channels; additionally, several new pathways, both normal and Umklapp, are also observed that can impact the thermal conductivity. We anticipate that our work will aid in the development of next-generation Peierls-Boltzmann transport simulations that can access all the pertinent normal mode scattering pathways from finite temperature *ab initio* simulations.

## Methods

### Real asymmetric normal mode amplitudes

Normal mode representations are not unique^1^; they only have to transform the coupled Hamiltonian to a set of independent harmonic oscillators^2^. Several descriptors of normal modes exist^1,2,55^ but not all of them are appropriate for probing the interactions among modes. Assuming that the equilibrium displacement of each atom *j* of the unit-cell *l* at any time *t* can be constructed as a sum of independent travelling waves ${u}_{\alpha }(j,l,t)=\sum _{{\bf{q}},p}\frac{1}{\sqrt{{N}_{u}{m}_{j}}}{Q}_{n}({\bf{q}},p,t){e}_{j,\alpha }({\bf{q}},p)\exp (i({\bf{q}}\,.\,{{\bf{r}}}_{l}))$^56^, the most frequently employed normal modes, which are complex, are given by:

2 $${Q}_{n}({\bf{q}},p,t)=\frac{1}{\sqrt{{N}_{u}}}\sum _{jl\alpha }\sqrt{{m}_{j}}\exp (-i({\bf{q}}\,.\,{{\bf{r}}}_{l})){e}_{j,\alpha }^{\ast }({\bf{q}},p)\frac{{\partial }^{n}}{\partial {t}^{n}}({u}_{\alpha }(j,l,t))={Q}_{n}^{\ast }(-{\bf{q}},p,t)$$

where *Q*_*n*_ represents the *n*^*th*^ derivative of the normal mode coordinates (for example, *n* = 0 corresponds to the standard positional normal mode coordinates), *α* is the spatial component of the displacement, and *p* and *e* represent the polarization index and the eigenvector of the dynamical matrix, respectively. The normal mode coordinate *Q*_*n*_ does not uniquely represent a wave traveling in the +**q** or −**q** direction, instead it denotes an average of both directions. Thus *Q*_*n*_ and the complex conjugate ${{\bf{Q}}}_{{n}}^{\ast }$, which are not independent of each other, cannot resolve an elementary wave separately into +**q** or −**q** directions^55^; the initial work to extract phonon-like interactions from atomistic simulations^49^ also noted the difficulty in unambiguously identifying N or U processes using the standard complex normal mode coordinates.

The heart of our approach rests on identifying and using a set of normal modes that can distinguish a right-going wave from a left-going wave with independent modal amplitudes and frequencies. To this end, we expand the atomic displacements as^55^:

3 $${u}_{\alpha }(j,l,t)=\frac{1}{\sqrt{{m}_{j}}}\sum _{{\bf{q}},p}{e}_{j,a}({\bf{q}},p)[{A}_{+}({\bf{q}},p,t)\exp (i({\bf{q}}\,.\,{{\bf{r}}}_{l}-w({\bf{q}},p)t))+{A}_{-}({\bf{q}},p,t)\exp (i({\bf{q}}\,.\,{{\bf{r}}}_{l}+w({\bf{q}},p)t))]$$

where ${A}_{+}({\bf{q}},p,t)$ and ${A}_{-}({\bf{q}},p,t)$ are the amplitudes of plane waves moving along +**q** and −**q** directions, respectively. After some algebra, the real displacement can be written as a superposition of cosine waves, each offset by an appropriate phase; it is given by:

4 $${u}_{\alpha }(j,l,t)=\sum _{{\bf{q}},p}\frac{1}{\sqrt{{m}_{j}}}A({\bf{q}},p,t)|{e}_{j,\alpha }({\bf{q}},p)|\cos ({\bf{q}}\,.\,{{\bf{r}}}_{l}-w({\bf{q}},p)t+\varphi ({\bf{q}},p)+{\varphi }_{j,\alpha }({\bf{q}},p))$$

where *A*(**q**, *p*) denotes the real amplitude (that can acquire negative values) of a wave travelling along the +**q** direction with an initial phase *ϕ,* and *A*(−**q**, *p*), which is not equal to *A*(**q**, *p*), delineates the amplitude of the wave traveling along the −**q** direction. The normal mode amplitude *A*(**q**, *p*, *t*) is real, unlike the complex *Q*(**q**, *p*, *t*), and more importantly, has the ability to resolve the +**q** and −**q** directions separately, making it  most apposite for probing phonon-like scattering mechanisms. The complex *Q*_*n*_ is related to the generalized mode amplitude *A*_*n*_ as:

5 $${A}_{n}^{2}({\bf{q}},p,t)=\frac{1}{{N}_{u}}[\frac{{Q}_{n}({\bf{q}},p,t)}{{w}^{n}({\bf{q}},p)}+i\frac{{Q}_{n+1}({\bf{q}},p,t)}{{w}^{n+1}({\bf{q}},p)}][\frac{{Q}_{n}^{\ast }({\bf{q}},p,t)}{{w}^{n}({\bf{q}},p)}-i\frac{{Q}_{n+1}^{\ast }({\bf{q}},p,t)}{{w}^{n+1}({\bf{q}},p)}]$$

where *N*_*u*_ is the number of unit cells. When *n* = {0, 1}, the conjugate variables that are used in the evaluation of *A*_*n*_ are the displacements and velocities; when the time dependence is also included, they are referred to as the real normal modes of the second kind, following Born and Huang^2^. The other combinations of *n* are new and may offer some advantages in certain situations. For example, the set of *n* = {1, 2}, which corresponds to velocities and accelerations, is better suited when there are uncertainties in ascertaining the equilibrium displacements but not for other dynamic variables. Now, with ${A}_{0}({\bf{q}},p,t)\equiv A({\bf{q}},p,t)$, the harmonic energy with the sign-preserved formulation can be written as:

6 $${H}_{2}({\bf{q}},p,t)={E}_{0}({\bf{q}},p,t)=\frac{1}{2}{N}_{u}{w}^{2}({\bf{q}},p){A}^{2}({\bf{q}},p,t)$$

Thus the modal energy is proportional to the square of the modal amplitude, and the total harmonic energy is simply the sum of *E*_0_(**q**, *p*, *t*) over all vibrational modes. In general, ${E}_{0}({\bf{q}},p,t)\ne {E}_{0}(-{\bf{q}},p,t)$ as +**q** and −**q** correspond to two different waves. It is also instructive to note that *Q*_*n*_ is a linear combination of *A*_*n*_(**q**, *p*, *t*) and *A*_*n*_(−**q**, *p*, *t*), and thus complex *Q*_*n*_(**q**, *p*, *t*) cannot be associated with a positive or negative **q** direction. The illuminating relationship below shows that the modal energies computed using *Q*_0_(**q**, *p*, *t*) and *Q*_1_(**q**, *p*, *t*) is actually the average of the energy associated with the modes traveling along opposite directions.

7 $$\begin{array}{rcl}{H}_{2}({\bf{q}},p,t) & = & \frac{1}{2}[{Q}_{1}({\bf{q}},p,t){Q}_{1}^{\ast }({\bf{q}},p,t)+{w}^{2}({\bf{q}},p){Q}_{0}({\bf{q}},p,t){Q}_{0}^{\ast }({\bf{q}},p,t)]\\  & = & [\frac{{E}_{0}({\bf{q}},p,t)+{E}_{0}(-{\bf{q}},p,t)}{2}]={H}_{2}(-{\bf{q}},p,t)\end{array}$$

Equations (6) and (7) bring out the essential difference between the real asymmetric (*A*) and the complex symmetric (*Q*) normal mode representations.

### Atomistic simulations

We perform atomistic simulations on a one-dimensional FPU^50–52^ lattice of *N* (100) atoms using periodic boundary conditions; we have verified that the dynamical behavior is ergodic and size-independent. The atoms are allowed to interact with only the first nearest neighbors, for both harmonic and anharmonic potentials. The anharmonic interactions are evaluated from the two-parameter (*σ*, *ε*) Lennard-Jones (LJ) potential^57^. For small displacements about the equilibrium position ${r}_{0}={2}^{1/6}\sigma$, the LJ interaction can be approximated as:

8 $${\rm{\Delta }}{U}_{LJ}(r)\approx \mathop{\overbrace{\frac{1}{2}C{(r-{r}_{0})}^{2}}}\limits^{{H}_{2}}+\mathop{\overbrace{\frac{1}{3}\alpha {(r-{r}_{0})}^{3}}}\limits^{{H}_{3}}+\mathop{\overbrace{\frac{1}{4}\beta {(r-{r}_{0})}^{4}}}\limits^{{H}_{4}}$$

where $C=36\sqrt[3]{4}\varepsilon {\sigma }^{-2}$. The FPU-*α* system interacts only through the cubic Hamiltonian (H_3_) while the FPU-*β* system interacts only with the quartic Hamiltonian (H_4_). The parameters *α* and *β* are such that they satisfy the Taylor approximation of the LJ interaction and they take the constant values $\alpha =-\,378\sqrt{2}\varepsilon {\sigma }^{-3}$ and $\beta =2226\sqrt[3]{2}\varepsilon {\sigma }^{-4}$. The lattice parameter *a* is set to the equilibrium separation *r*_0_. All the results that are reported are in standard reduced units^57^ using atomic mass *m*, length *σ* and energy *ε*; for example, the reduced time that is reported is given by $\tau {(m{\sigma }^{2}/\varepsilon )}^{-1/2}$, where *τ* is the physical time. We further note here that a large number of investigations, both theoretical and numerical, have been reported on FPU lattices since the seminal discovery of recurrence by Fermi, Pasta and Ulam^58^. It is now known that above a stochastic threshold, equipartition of energy among the different modes can be enforced in FPU lattices^50^. Recent theoretical work^59^ also shows that even the original FPU lattices studied by Fermi, Pasta and Ulam can be thermalized from higher order resonant interactions for arbitrarily small linearity. As elucidated earlier, the chosen system length and other parameters allow complete thermalization and equipartition of energy among the normal modes for the FPU lattices that are considered in this work.

Two kinds of simulations are performed in this investigation. To probe the normal mode interactions (as shown in Fig. 1), the atoms which are initially placed at the equilibrium positions (**r**_*j*_), are perturbed by a normal mode with a direction-dependent wave-vector **q** (with an excess energy *E*_*x*_). This is achieved by perturbing all the atoms (*j*) by an initial displacement and velocity given by:

9 $${u}_{j}({\bf{q}})=\frac{1}{\sqrt{m}}A({\bf{q}})\cos ({\bf{q}}\,.{{\bf{r}}}_{j}+\varphi ({\bf{q}}))$$

10 $${v}_{j}({\bf{q}})=\frac{1}{\sqrt{m}}A({\bf{q}})w({\bf{q}})\sin ({\bf{q}}\,.{{\bf{r}}}_{j}+\varphi ({\bf{q}}))$$

where the initial phase *ϕ*(**q**) is randomly sampled from 0 to 2π. The initial velocities are required to perturb the modes in the chosen direction; otherwise, if only the displacements are initialized with zero initial velocities, normal modes would be excited along both +**q** and −**q** directions, thereby creating a standing wave. The rate of change of modal amplitudes is assumed to be small relative to the change in the time phase factor under the assumption that the normal mode lifetimes span several vibrational time periods^43^. The external perturbation, which corresponds to an excitation energy *E*_*x*_ as depicted in Fig. 4, is used to generate a phonon mode with a direction-dependent wave-vector **q**. The system then responds to the external perturbation and several additional normal modes are generated. The external perturbation is then repeated for different **q** vectors. Figure 1 shows both the modes that are perturbed (along the *y*-axis) and the modes that are generated or excited (along the *x*-axis).

Newton’s equations of motion are integrated with the symplectic velocity-Verlet algorithm using a timestep of 0.0002 and the normal modes are identified after 0.4 time units. This brief time period allows detection of the primary interactions among the normal modes arising directly from the external perturbation. Thus, the chosen observation window corresponds to the order of τ_*D*_, the Debye timescale (0.30 time units) defined as 2*a*/*c*, where *a* is the lattice spacing and *c* is the speed of sound. The results portrayed in Figs. 1 and 5 are realized through the aforesaid perturbation protocol. The Fourier transform data presented in Fig. 3 is obtained using a similar approach, but with a timestep of 0.001, and the mode energies, evaluated every 0.03 time units, are collected for a total of 300 time units. Temperature is not controlled in the perturbation protocol.

Ensemble and time-averaged simulations, results of which are depicted in Fig. 2, are performed at a finite temperature, however, without using an external thermal bath or rescaling. Ensemble averages are computed by varying the initial conditions (positions and velocities) and taking an average over several sets of initial conditions. Starting with all the atoms at their equilibrium positions – this corresponds to a state with the minimum potential energy – the initial velocities of the atoms are sampled from the Maxwell-Boltzmann distribution with a mean kinetic energy of *k*_*B*_*T* per atom. The system is then allowed to evolve in an NVE ensemble and letting the energy to redistribute between the potential and kinetic degrees of freedom. The system thus is driven to an equilibrium state at a temperature *T* with an average potential and kinetic energy of *k*_*B*_*T*/2, respectively, per atom. We have verified that the system reaches thermal equilibrium obeying equipartition of energy among different phonon modes without giving preferential excitation to any modes. The FPU lattices are first equilibrated for 15,000 time steps at a temperature of 0.01 with a time step of 0.002 in reduced units; equilibrium data is then collected for a time period of 30 units. We also adopt the same numerical procedure for graphene^60^, which is discussed in the Supplemental Information.

## Supplementary information

Supplemental Information Phonon Normal Modes Raj Eapen

## Data Availability

The datasets generated during and/or analysed during the current study are available from the corresponding author on reasonable request.
