# Supplementary material for: Deducing Phonon Scattering from Normal Mode Excitations
Source: Sci Rep. 2019 May 30;9:7982. doi: 10.1038/s41598-019-43306-3 (PMC6538846; doi:10.1038/s41598-019-43306-3)
Supplement: Supplementary file 1 — Supplemental Information Phonon Normal Modes Raj Eapen [file 41598_2019_43306_MOESM1_ESM.pdf]

Supplemental Information

Deducing Phonon Scattering from Normal Mode Excitations

Anant Raj and Jacob Eapen*

Department of Nuclear Engineering
North Carolina State University, Raleigh, NC 27695, USA
*jacob.eapen@ncsu.edu

A: Protocol for generating the excited modes shown in Figure 1 of the main text

The methodology for determining the excited normal modes through the perturbation of a single normal mode (as shown in Figure 1 of the main text) is delineated below. We will illustrate the method using a FPU-α system with N atoms (N =100) having a lattice constant a. The allowed wave vectors in the first Brillouin zone for this system are given by:

$$q = \frac{2r}{N}\left(\frac{\pi}{a}\right); r \in \mathbb{Z}; -\left(\frac{\pi}{a}\right) < q \le \left(\frac{\pi}{a}\right) \tag{S1}$$

Step 1: Start with all the atoms at rest in their equilibrium positions.

Step 2: Chose a wavevector q as given by Eqn. (S1).

Step 3: Perturb the mode q with energy E_x by setting the displacements and velocities of every atom (j) of the system as:

$$\left.\begin{aligned} u_j(q) &= \frac{1}{\sqrt{m}} A(q)\cos\left(qr_j + \phi(q)\right) \\ v_j(q) &= \frac{1}{\sqrt{m}} A(q)w(q)\sin\left(qr_j + \phi(q)\right) \end{aligned}\right| \begin{aligned} A(q) &= \sqrt{\frac{2E_x}{Nw^2(q)}} \\ j &\in [1,N]; \phi \in [0,2\pi) \end{aligned} \tag{S2}$$

Step 4: Allow the system to evolve in time. The externally perturbed mode q interacts and exchanges energy with other modes, and in the process excites new normal modes q' (primary excitations). The newly excited modes can then undergo further interactions to generate secondary excitations.

Step 5: Measure the average energy associated with each excited normal mode over a short time interval t_0 (0.4 time units for FPU systems) following the initial perturbation. The energy is measured as:

$$E\left(q',\phi(q)\right) = \frac{1}{t_0}\int_o^{t_0} E\left(q',\phi(q),t\right)dt \tag{S3}$$

The short time interval of observation (t_0) is to minimize the occurrences of the secondary excitations thereby ensuring that only the dominant primary excitations are recorded. If more time is allowed, both primary and secondary excitations will be generated.

Step 6: Repeat **Steps 3 through 5** for P independent simulations with different initial phases $\phi(q)$ (see Eqn. (S2)), randomly selected from zero to 2π. Each modal energy that is excited is then averaged over several values of the initial phase of the perturbed mode as shown by:

$$E(q') = \frac{1}{P}\sum_{i=1}^{P} E\left(q', \phi_i(q)\right) \tag{S4}$$

Step 7: Plot the energies of all the modes (q') excited by the perturbed mode q, as shown in Figure 1 of the main text. Note that the perturbed mode q varies along the y-axis, while the corresponding excited modes are plotted along the x-axis.

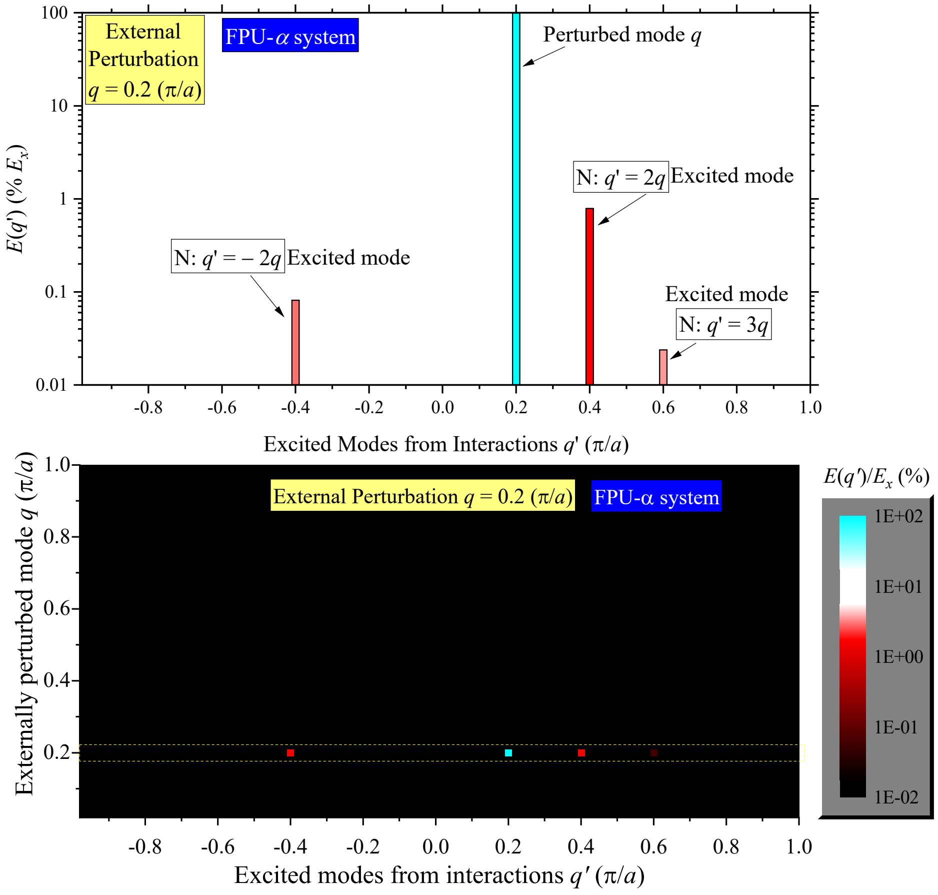

Fig. S1. (Top) The excited modes (q') and the corresponding modal energies following the initial perturbation of the mode q = 0.2 (π/a) for the FPU-α system. (Bottom) The corresponding excited modes in the layout of Figure 1 of the main text from the same perturbation (energies are now depicted by the color scale) are marked between the dotted yellow lines.

As an example, consider the perturbation of the mode q = 0.2 (π/a). The excited modes (q') and the associated modal energies following the external perturbation averaged over P=100 independent simulations at time t_0 = 0.4 time units are shown in Fig. S1 (top) for FPU-α system.

As expected, the bulk of the energy is contained in the mode q that is initially perturbed, while a small fraction of the energy is transferred to the excited modes $2q$ and $-2q$, which are the primary excitations. The $2q$ mode is produced by an N-type combination from the mode q while the $-2q$ mode is produced by an N-type simultaneous creation event (see the main text for details). Interestingly, even in this small time window, we observe a very feeble $3q$ mode, which is a secondary excitation.

All the modes (q') excited by the initial perturbation correspond to a single horizontal line in the layout of Figure1 of the main text. The corresponding line of exited modes (q') from the initial perturbation of q = 0.2 (π/a) mode is shown in Fig. S1 (bottom), marked between the two dotted yellow lines. Each of the four peaks in Fig. S1 (top) appear as points in Fig. S1 (bottom); the corresponding modal energies are depicted by the color scale.

Step 8: Repeat **Steps 1 through 7** for all wavevectors in the first Brillouin zone (given by Eqn. (S1)). Each additional perturbation will generate more excited modes. As an example, the excited modes from perturbing q = 0.6 (π/a) are shown in Fig. S2. Since $2q$ is greater than π/a, the primary excitations ($2q$ and $-2q$) get reflected at the Brillouin zone boundary to produce U-type interactions. The corresponding line of excited modes (q') is shown in Fig. S2 (bottom), marked between the two dotted yellow lines. Note that the excited modes from the perturbation of q = 0.2 (π/a) are also shown.

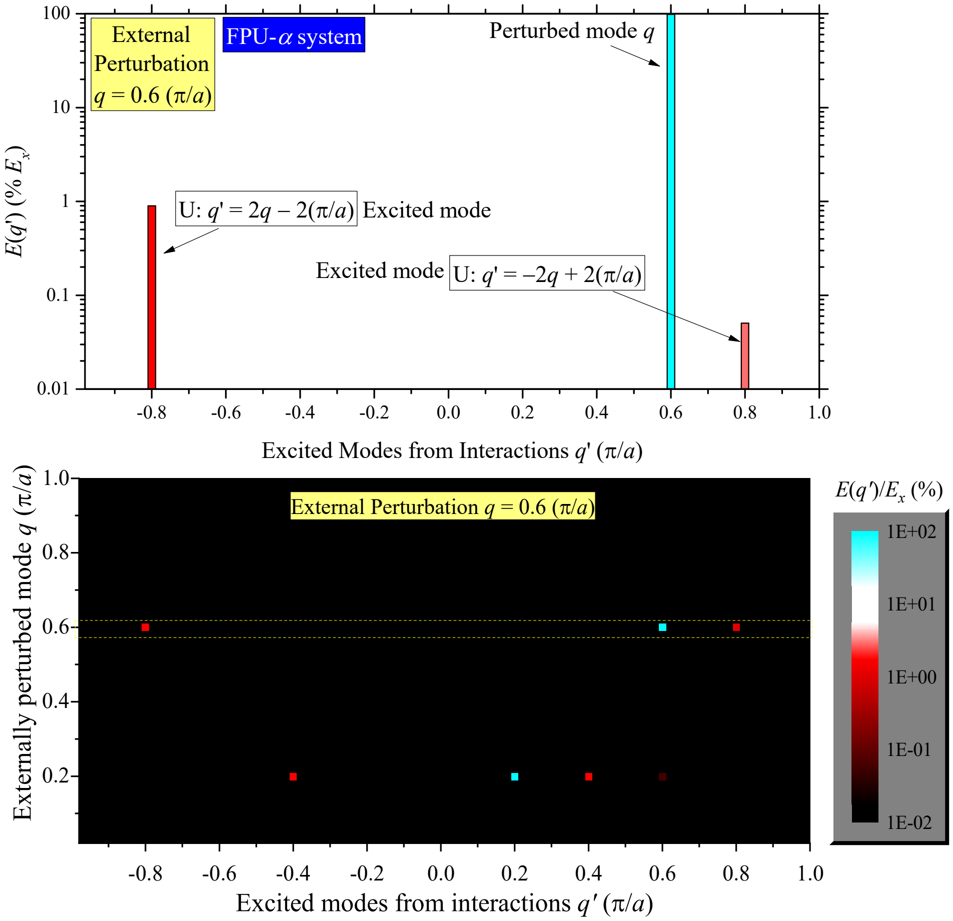

Fig. S2. (Top) The excited modes (q') and the corresponding modal energies following the initial perturbation of the mode q = 0.6 (π/a) for the FPU-α system. (Bottom) The corresponding excited modes in the layout of Figure 1 of the main text from the same perturbation (energies are now depicted by the color scale) are marked between the dotted yellow lines. The excited modes from the perturbation of q = 0.2 (π/a) are also shown.

The plots in Fig. S3 show the population of the excited modes by perturbing different wave vectors. Repeating the same process for all possible wave vectors in the first Brillouin zone, as given by Eqn. (S1), we generate Fig. S4, which is the same as Figure 1 of the main text.

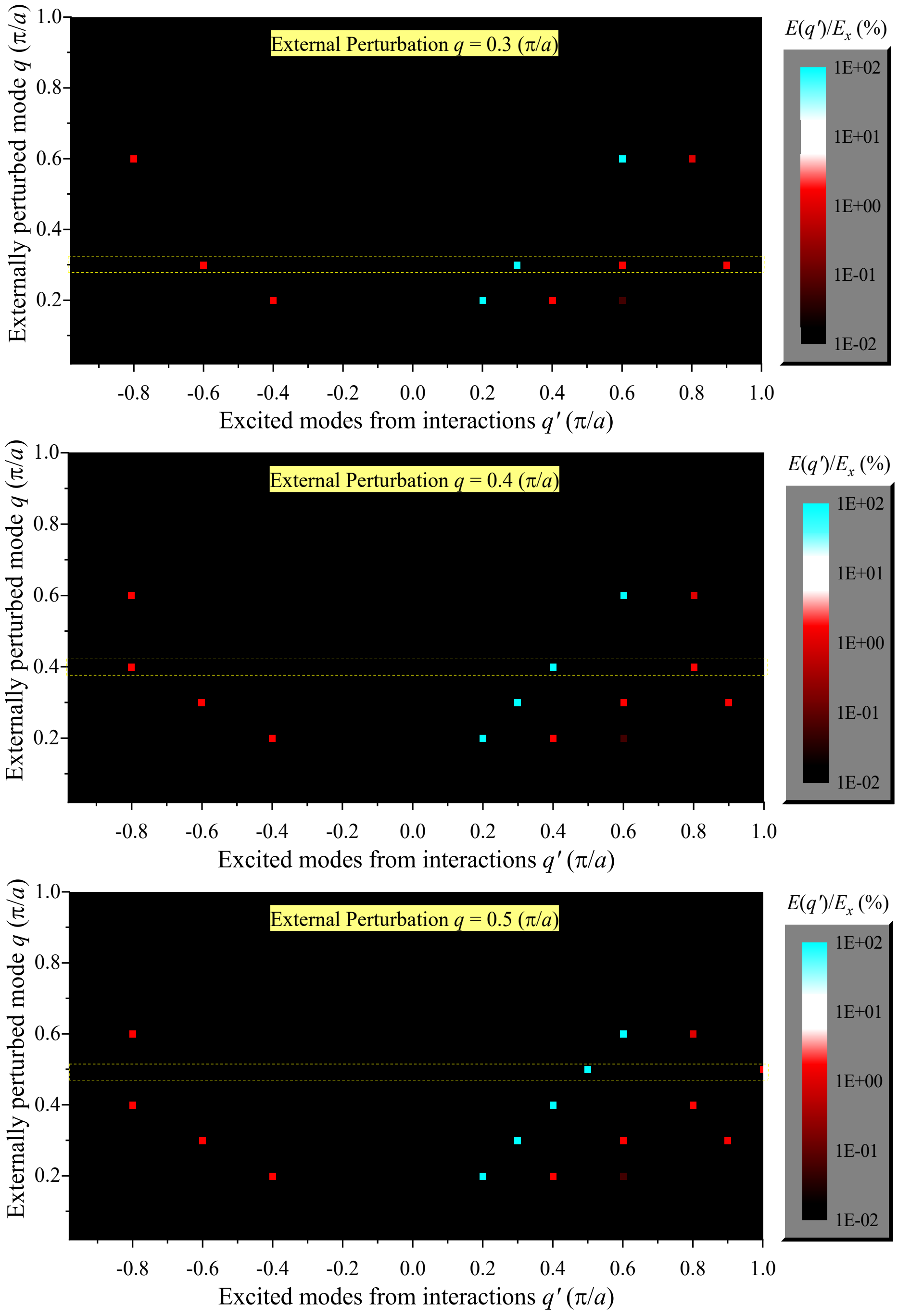

Fig S3: Populating the excited modes for three successive iterations of **Steps 1 through 7** with different wavevectors ($q = 0.3$ (π/a), $q = 0.4$ (π/a) and $q = 0.5$ (π/a)) for the FPU-α system.

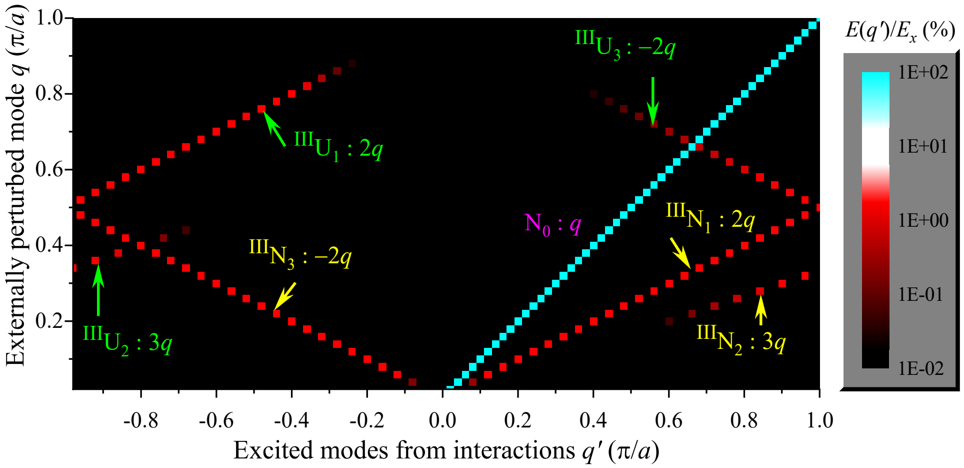

Fig. S4: Repeating **Steps 1 through 7** for all possible wave vectors in the first Brillouin zone, as given by Eqn. (S1), to generate Figure 1 (top) of the main text.

B: Normal modes of graphene using optimized Tersoff potential

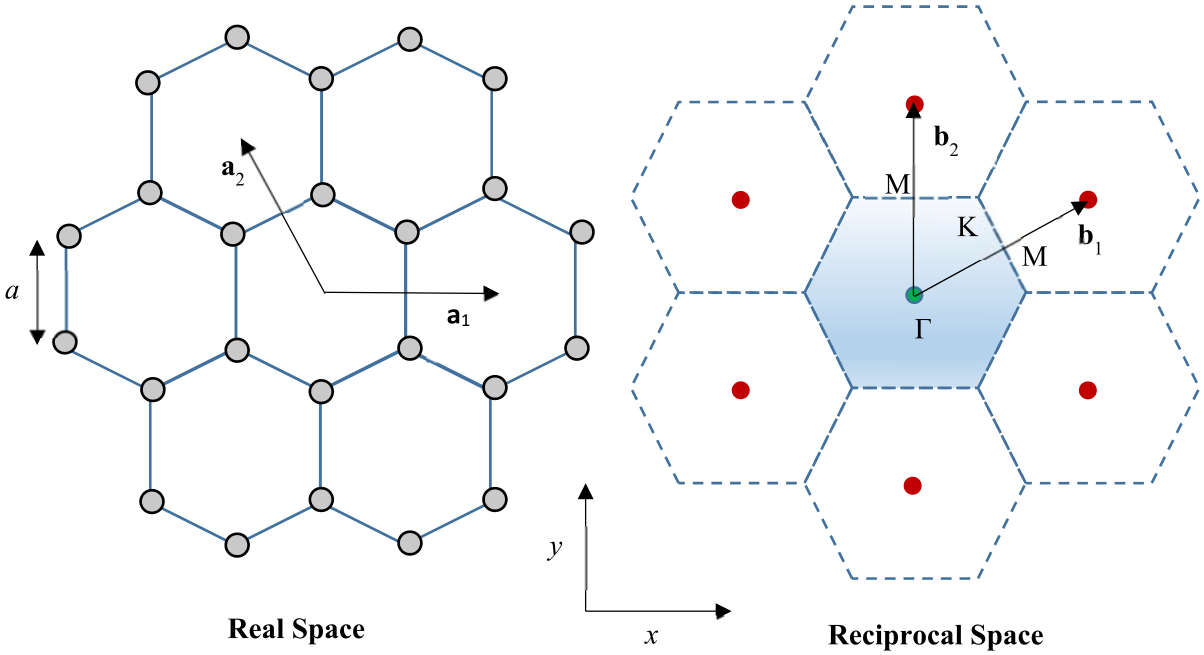

Fig. S5: The real space (left) and reciprocal space (right) of graphene. The x-axis and the y-axis are along the zigzag and armchair directions, respectively. The unit vectors are given by $\mathbf{a}_1$ and $\mathbf{a}_2$, whereas the corresponding reciprocal space vectors are delineated by $\mathbf{b}_1$ and $\mathbf{b}_2$.

Carbon atoms are arranged in a triangular lattice in graphene with a two-atom basis as shown in Fig. S5. Atomistic simulations are performed with 60 unit-cells along both the unit vector directions constituting a

total of 7200 carbon atoms. Atoms interact through the Tersoff potential[1] optimized by Lindsay and Broido[2]. Periodic boundary conditions are applied along the unit vector directions. Equilibration and equipartition are established using the approach devised for the FPU lattices (see Methods section in the main text). The initial velocities are sampled from a Maxwell-Boltzmann distribution with a kinetic energy of 30 k_B per atom (corresponding to a temperature of 30 *K*). The system is then allowed to relax for 200 *ps* with an integration timestep of 1 *fs* in an NVE ensemble during which the excess kinetic energy is redistributed between the potential and kinetic degrees of freedom. After equilibration, the system attains a temperature of 30 *K*. Simulations with the optimized parameters have predicted both the longitudinal and flexural acoustic branches with acceptable accuracy. We show the phonon dispersion curves[3] in Fig. S6.

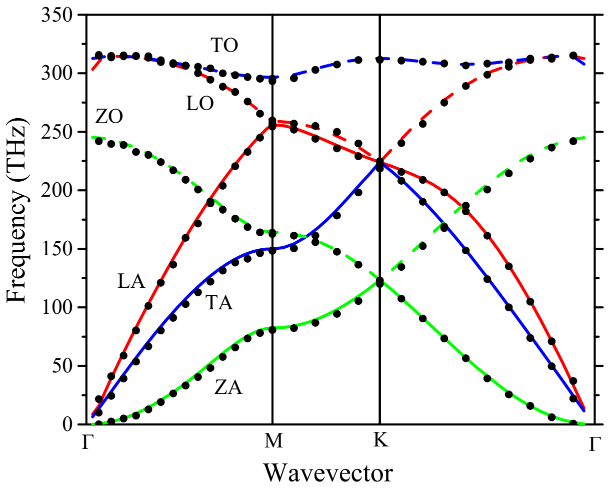

Fig. S6. Phonon dispersion curves (solid lines) for graphene along the high symmetry directions. The reference data of Lindsay and Broido[2] are over-laid as solid circles.

C: LA and LO excitations in graphene

The modal excitation methodology described previously is employed for graphene too. In Fig. S7, the *x*-axis denotes the wave vector of the LA mode that is initially perturbed while the excited modes are represented along the *y*-axis (in both right and left panels). The magnitude of the mode is represented by the corresponding modal energy, which is given in log scale. The brightest mode ${}^{LA}N_0$ (left panel) corresponds to the initially perturbed mode. As with the FPU lattices, both N and U type processes can be identified. The most prominent LA branch corresponds to a Class I three-phonon merging interaction given by: $\mathbf{q}_{LA} + \mathbf{q}_{LA} \to 2\mathbf{q}_{LA}$. Interestingly, the U processes are weak for the excited LA modes but prominent for the excited LO modes (right panel). We can explain this by noting that when the excited mode $2\mathbf{q}_{LA}$ becomes large enough, it crosses the zone boundary and gets reflected as a LO mode. This U interaction, which can be expressed as: $\mathbf{q}_{LA} + \mathbf{q}_{LA} \to 2\mathbf{q}_{LA} - \mathbf{g}_{\Gamma M} = \mathbf{q}'_{LO}$ is more conspicuous than the $\mathbf{q}'_{LA}$ mode that emerges from the interaction: $\mathbf{q}_{LA} + \mathbf{q}_{LA} \to 2\mathbf{q}_{LA} - \mathbf{g}_{\Gamma M} = \mathbf{q}'_{LA}$. Since swapping to the LO branch gives a smoother path, as opposed to a sharp turn at the M point for the LA branch (see Fig. S8), the reflected LO branch appears more prominent. Almost equally prominent is the backscattering $-\mathbf{q}$ mode for both LA and LO branches; the origin of these modes is discussed in the next section.

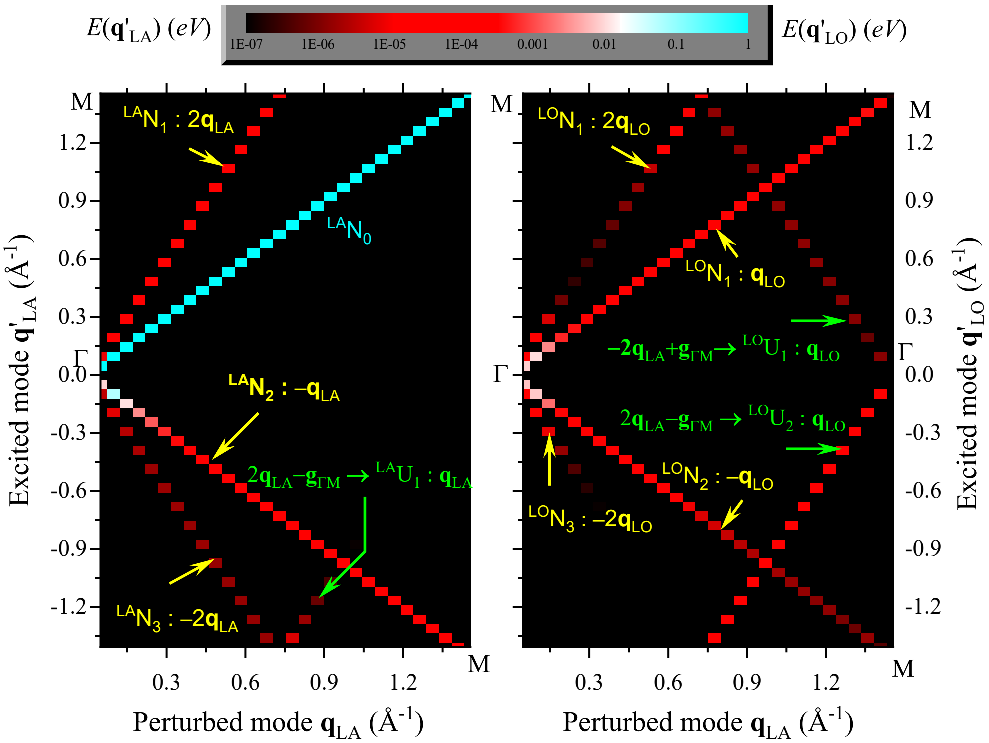

Fig. S7: (left) Excited LA and (right) LO normal modes along the ΓM direction detected during the first 100 fs following the perturbation of a single LA mode with 1 eV. The excited modes are generated with the same protocol outlined in Section A.

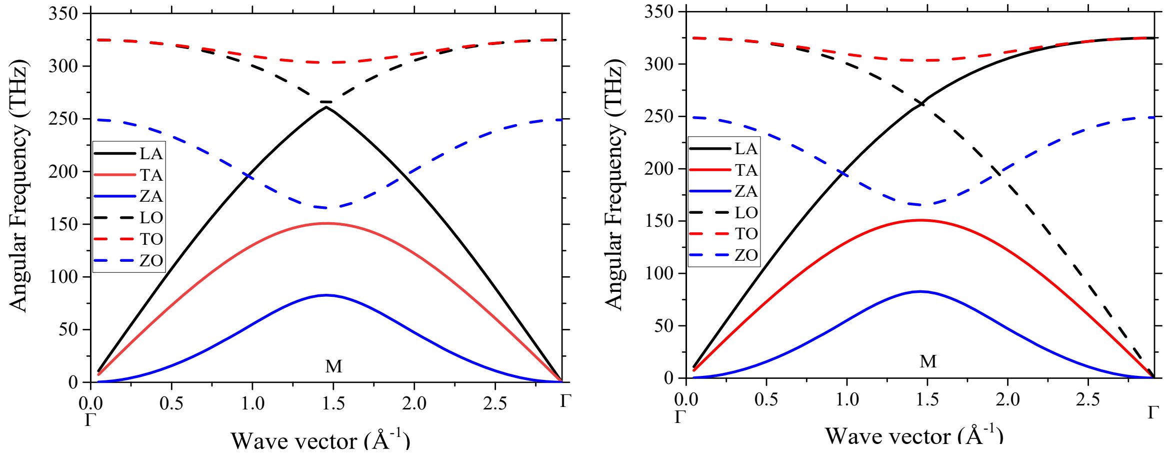

Fig. S8: Extension of the normal mode dispersion for graphene along the ΓMΓ direction. (left) The branches are simply reflected about the M point, (right) LA and LO branches are swapped across the M point.

D: Fluctuations of harmonic energy in graphene

Analysis of the time-varying Hamiltonians shows that the negative $-\mathbf{q}_{LA}$ mode emerges from a four-phonon simultaneous creation process: $\mathbf{q}_{LA} \rightarrow \mathbf{q}_{LA} + \mathbf{q}_{LA} + \left(-\mathbf{q}_{LA}\right)$, which is similar to that in the FPU-β lattice with only quartic potential. The oscillating harmonic Hamiltonian provides the additional energy that is needed

for this four-phonon creation process, which otherwise is forbidden with constant anharmonic Hamiltonians. Frequency analysis, performed by taking the Fourier transform of the deviation of the energy associated with each mode, rules out the possibility of a three-phonon splitting event: $\mathbf{q}_{LA} \rightarrow 2\mathbf{q}_{LA} + \left(-\mathbf{q}_{LA}\right)$.

We note here that the key objective of the current work is the extraction of normal mode interactions directly from atomistic dynamics. Of particular interest are the inherent fluctuations in harmonic and anharmonic Hamiltonians, and the large number of scattering pathways that are engendered by the oscillatory Hamiltonians. The somewhat more complex observations on graphene reinforce the salient characteristics of the normal mode interactions drawn out with the FPU lattices.

E: Derivation of the cubic Hamiltonian:

The general solution for the α component of the displacement of the jth atom of the lth unit-cell can be written as:

$$u_\alpha\left(j,l,t\right) = \sum_{\mathbf{q},p} \frac{e_{j,\alpha}\left(\mathbf{q},p\right)}{\sqrt{m_j}} \left[A_+\left(\mathbf{q},p,t\right)\exp\left(i\left(\mathbf{q}.\mathbf{r}_l - w\left(\mathbf{q},p\right)t\right)\right) + A_-\left(\mathbf{q},p,t\right)\exp\left(i\left(\mathbf{q}.\mathbf{r}_l + w\left(\mathbf{q},p\right)t\right)\right)\right] \quad \text{(S5)}$$

where the displacement u is expressed as a superposition of left moving and right moving traveling waves.

The cubic Hamiltonian can be formally expressed as[4]:

$$H_3\left(t\right) = \frac{1}{3!} \sum_{j'',l'',\gamma} \sum_{j',l',\beta} \sum_{j,l,\alpha} \sum_{\mathbf{q}_1,p_1} \sum_{\mathbf{q}_2,p_2} \sum_{\mathbf{q}_3,p_3} G_{j'',l'',\gamma,j',l',\beta,j,l,\alpha}\, u_\alpha^{\mathbf{q}_1 p_1}\left(j,l,t\right) u_\beta^{\mathbf{q}_2 p_2}\left(j',l',t\right) u_\gamma^{\mathbf{q}_3 p_3}\left(j'',l'',t\right) \quad \text{(S6)}$$

where G is the cubic force constant matrix and $u_\alpha^{\mathbf{q}_1 p_1}\left(j,l,t\right)$ is the displacement due to the wave travelling along $+\mathbf{q}_1$ direction with mode index p_1. The summation is carried over all possible three-atom interactions (j, j' and j'') resulting from all possible normal modes ($\mathbf{q}_i$,p_i). The cubic Hamiltonian can now be expanded as:

$$H_3\left(t\right) = \sum_{j,j',j'',\alpha,\beta,\gamma} \sum_{p_1,p_2,p_3} \sum_{\mathbf{q}_1,\mathbf{q}_2,\mathbf{q}_3} \sum_{l,l',l''} \frac{G_{j'',l'',\gamma,j',l',\beta,j,l,\alpha}}{3!} \left(\begin{bmatrix} e_{j,\alpha}\left(\mathbf{q}_1,p_1\right) A_+\left(\mathbf{q}_1,p_1,t\right)\exp\left(i\left(\mathbf{q}_1.\mathbf{r}_l - w\left(\mathbf{q}_1,p_1\right)t\right)\right) \\ +e^*_{j,\alpha}\left(\mathbf{q}_1,p_1\right) A^*_+\left(\mathbf{q}_1,p_1,t\right)\exp\left(-i\left(\mathbf{q}_1.\mathbf{r}_l - w\left(\mathbf{q}_1,p_1\right)t\right)\right) \end{bmatrix} \begin{bmatrix} e_{j',\beta}\left(\mathbf{q}_2,p_2\right) A_+\left(\mathbf{q}_2,p_2,t\right)\exp\left(i\left(\mathbf{q}_2.\mathbf{r}_{l'} - w\left(\mathbf{q}_2,p_2\right)t\right)\right) \\ +e^*_{j',\beta}\left(\mathbf{q}_2,p_2\right) A^*_+\left(\mathbf{q}_2,p_2,t\right)\exp\left(-i\left(\mathbf{q}_2.\mathbf{r}_{l'} - w\left(\mathbf{q}_2,p_2\right)t\right)\right) \end{bmatrix} \begin{bmatrix} e_{j'',\gamma}\left(\mathbf{q}_3,p_3\right) A_+\left(\mathbf{q}_3,p_3,t\right)\exp\left(i\left(\mathbf{q}_3.\mathbf{r}_{l''} - w\left(\mathbf{q}_3,p_3\right)t\right)\right) \\ +e^*_{j'',\gamma}\left(\mathbf{q}_3,p_3\right) A^*_+\left(\mathbf{q}_3,p_3,t\right)\exp\left(-i\left(\mathbf{q}_3.\mathbf{r}_{l''} - w\left(\mathbf{q}_3,p_3\right)t\right)\right) \end{bmatrix} \right) \quad \text{(S7)}$$

Expanding the product:

$$H_3(t)=\sum_{j,j',j'',\alpha,\beta,\gamma}\sum_{p_1,p_2,p_3}\sum_{\mathbf{q}_1,\mathbf{q}_2,\mathbf{q}_3}\sum_{l,l',l''}\frac{G_{j'',l'',\gamma,j',l',\beta,j,l,\alpha}}{3!}\left(\begin{array}{l}\begin{bmatrix}e_{j,\alpha}(\mathbf{q}_1,p_1)A_+(\mathbf{q}_1,p_1,t)\exp\left(i\left(\mathbf{q}_1.\mathbf{r}_l-w(\mathbf{q}_1,p_1)t\right)\right)\\ e_{j',\beta}(\mathbf{q}_2,p_2)A_+(\mathbf{q}_2,p_2,t)\exp\left(i\left(\mathbf{q}_2.\mathbf{r}_{l'}-w(\mathbf{q}_2,p_2)t\right)\right)\\ e_{j'',\gamma}(\mathbf{q}_3,p_3)A_+(\mathbf{q}_3,p_3,t)\exp\left(i\left(\mathbf{q}_3.\mathbf{r}_{l''}-w(\mathbf{q}_3,p_3)t\right)\right)\end{bmatrix}+CC\\ +\begin{bmatrix}e_{j,\alpha}(\mathbf{q}_1,p_1)A_+(\mathbf{q}_1,p_1,t)\exp\left(i\left(\mathbf{q}_1.\mathbf{r}_l-w(\mathbf{q}_1,p_1)t\right)\right)\\ e_{j',\beta}(\mathbf{q}_2,p_2)A_+(\mathbf{q}_2,p_2,t)\exp\left(i\left(\mathbf{q}_2.\mathbf{r}_{l'}-w(\mathbf{q}_2,p_2)t\right)\right)\\ e^*_{j'',\gamma}(\mathbf{q}_3,p_3)A^*_+(\mathbf{q}_3,p_3,t)\exp\left(-i\left(\mathbf{q}_3.\mathbf{r}_{l''}-w(\mathbf{q}_3,p_3)t\right)\right)\end{bmatrix}+CC\\ +\begin{bmatrix}e_{j,\alpha}(\mathbf{q}_1,p_1)A_+(\mathbf{q}_1,p_1,t)\exp\left(i\left(\mathbf{q}_1.\mathbf{r}_l-w(\mathbf{q}_1,p_1)t\right)\right)\\ e^*_{j',\beta}(\mathbf{q}_1,p_1)A^*_+(\mathbf{q}_2,p_2,t)\exp\left(-i\left(\mathbf{q}_2.\mathbf{r}_{l'}-w(\mathbf{q}_2,p_2)t\right)\right)\\ e_{j'',\gamma}(\mathbf{q}_3,p_3)A_+(\mathbf{q}_3,p_3,t)\exp\left(i\left(\mathbf{q}_3.\mathbf{r}_{l''}-w(\mathbf{q}_3,p_3)t\right)\right)\end{bmatrix}+CC\\ +\begin{bmatrix}e_{j,\alpha}(\mathbf{q}_1,p_1)A_+(\mathbf{q}_1,p_1,t)\exp\left(i\left(\mathbf{q}_1.\mathbf{r}_l-w(\mathbf{q}_1,p_1)t\right)\right)\\ e^*_{j',\beta}(\mathbf{q}_1,p_1)A^*_+(\mathbf{q}_2,p_2,t)\exp\left(-i\left(\mathbf{q}_2.\mathbf{r}_{l'}-w(\mathbf{q}_2,p_2)t\right)\right)\\ e^*_{j'',\gamma}(\mathbf{q}_3,p_3)A^*_+(\mathbf{q}_3,p_3,t)\exp\left(-i\left(\mathbf{q}_3.\mathbf{r}_{l''}-w(\mathbf{q}_3,p_3)t\right)\right)\end{bmatrix}+CC\end{array}\right) \quad \text{(S8)}$$

where CC denotes the complex conjugate term. Rearranging and simplifying:

$$\begin{aligned}H_3(t)=&\sum_{j,j',j'',\alpha,\beta,\gamma}\sum_{p_1,p_2,p_3}\sum_{\mathbf{q}_1,\mathbf{q}_2,\mathbf{q}_3}\sum_{l}\frac{G_{j'',l'',\gamma,j',l',\beta,j,l,\alpha}}{3!}\left(\begin{bmatrix}e_{j,\alpha}(\mathbf{q}_1,p_1)e_{j',\beta}(\mathbf{q}_2,p_2)e_{j'',\gamma}(\mathbf{q}_3,p_3)\sum_{l'}\exp\left(i\left(\mathbf{q}_2.\mathbf{r}_{l'-l}\right)\right)\\ A_+(\mathbf{q}_1,p_1,t)A_+(\mathbf{q}_2,p_2,t)A_+(\mathbf{q}_3,p_3,t)\sum_{l''}\exp\left(i\left(\mathbf{q}_3.\mathbf{r}_{l''-l}\right)\right)\\ \exp\left(-i\left(\left[w(\mathbf{q}_1,p_1)+w(\mathbf{q}_2,p_2)+w(\mathbf{q}_3,p_3)\right]t\right)\right)\\ \exp\left(i\left((\mathbf{q}_1+\mathbf{q}_2+\mathbf{q}_3).\mathbf{r}_l\right)\right)\end{bmatrix}+CC\right)\\ &+\sum_{j,j',j'',\alpha,\beta,\gamma}\sum_{p_1,p_2,p_3}\sum_{\mathbf{q}_1,\mathbf{q}_2,\mathbf{q}_3}\sum_{l}\frac{G_{j'',l'',\gamma,j',l',\beta,j,l,\alpha}}{3!}\left(\begin{bmatrix}e_{j,\alpha}(\mathbf{q}_1,p_1)e_{j',\beta}(\mathbf{q}_2,p_2)e^*_{j'',\gamma}(\mathbf{q}_3,p_3)\sum_{l'}\exp\left(i\left(\mathbf{q}_2.\mathbf{r}_{l'-l}\right)\right)\\ A_+(\mathbf{q}_1,p_1,t)A_+(\mathbf{q}_2,p_2,t)A^*_+(\mathbf{q}_3,p_3,t)\sum_{l''}\exp\left(i\left(-\mathbf{q}_3.\mathbf{r}_{l''-l}\right)\right)\\ \exp\left(-i\left(\left[w(\mathbf{q}_1,p_1)+w(\mathbf{q}_2,p_2)-w(\mathbf{q}_3,p_3)\right]t\right)\right)\\ \exp\left(i\left((\mathbf{q}_1+\mathbf{q}_2-\mathbf{q}_3).\mathbf{r}_l\right)\right)\end{bmatrix}+CC\right)\\ &+\sum_{j,j',j'',\alpha,\beta,\gamma}\sum_{p_1,p_2,p_3}\sum_{\mathbf{q}_1,\mathbf{q}_2,\mathbf{q}_3}\sum_{l}\frac{G_{j'',l'',\gamma,j',l',\beta,j,l,\alpha}}{3!}\left(\begin{bmatrix}e_{j,\alpha}(\mathbf{q}_1,p_1)e^*_{j',\beta}(\mathbf{q}_2,p_2)e_{j'',\gamma}(\mathbf{q}_3,p_3)\sum_{l'}\exp\left(i\left(-\mathbf{q}_2.\mathbf{r}_{l'-l}\right)\right)\\ A_+(\mathbf{q}_1,p_1,t)A^*_+(\mathbf{q}_2,p_2,t)A_+(\mathbf{q}_3,p_3,t)\sum_{l''}\exp\left(i\left(\mathbf{q}_3.\mathbf{r}_{l''-l}\right)\right)\\ \exp\left(-i\left(\left[w(\mathbf{q}_1,p_1)-w(\mathbf{q}_2,p_2)+w(\mathbf{q}_3,p_3)\right]t\right)\right)\\ \exp\left(i\left((\mathbf{q}_1-\mathbf{q}_2+\mathbf{q}_3).\mathbf{r}_l\right)\right)\end{bmatrix}+CC\right)\\ &+\sum_{j,j',j'',\alpha,\beta,\gamma}\sum_{p_1,p_2,p_3}\sum_{\mathbf{q}_1,\mathbf{q}_2,\mathbf{q}_3}\sum_{l}\frac{G_{j'',l'',\gamma,j',l',\beta,j,l,\alpha}}{3!}\left(\begin{bmatrix}e_{j,\alpha}(\mathbf{q}_1,p_1)e^*_{j',\beta}(\mathbf{q}_2,p_2)e^*_{j'',\gamma}(\mathbf{q}_3,p_3)\sum_{l'}\exp\left(i\left(-\mathbf{q}_2.\mathbf{r}_{l'-l}\right)\right)\\ A_+(\mathbf{q}_1,p_1,t)A^*_+(\mathbf{q}_2,p_2,t)A^*_+(\mathbf{q}_3,p_3,t)\sum_{l''}\exp\left(i\left(-\mathbf{q}_3.\mathbf{r}_{l''-l}\right)\right)\\ \exp\left(-i\left(\left[w(\mathbf{q}_1,p_1)-w(\mathbf{q}_2,p_2)-w(\mathbf{q}_3,p_3)\right]t\right)\right)\\ \exp\left(i\left((\mathbf{q}_1-\mathbf{q}_2-\mathbf{q}_3).\mathbf{r}_l\right)\right)\end{bmatrix}+CC\right)\end{aligned} \quad \text{(S9)}$$

Changing indices, $l'-l=h'$ and $l''-l=h''$:

$$H_3(t)=\sum_{j,j',j'',\alpha,\beta,\gamma}\sum_{p_1,p_2,p_3}\sum_{\mathbf{q}_1,\mathbf{q}_2,\mathbf{q}_3}\left(\begin{bmatrix}\sum_{h'h''}\frac{G_{j'',h'',\gamma,j',h',\beta,j,l,\alpha}}{3!}\exp\left(i\left(\mathbf{q}_2.\mathbf{r}_{h'}\right)\right)\exp\left(i\left(\mathbf{q}_3.\mathbf{r}_{h''}\right)\right)\\ e_{j,\alpha}(\mathbf{q}_1,p_1)e_{j',\beta}(\mathbf{q}_2,p_2)e_{j'',\gamma}(\mathbf{q}_3,p_3)\\ A_+(\mathbf{q}_1,p_1,t)A_+(\mathbf{q}_2,p_2,t)A_+(\mathbf{q}_3,p_3,t)\\ \exp\left(-i\left(\left[w(\mathbf{q}_1,p_1)+w(\mathbf{q}_2,p_2)+w(\mathbf{q}_3,p_3)\right]t\right)\right)\\ \sum_l\exp\left(i\left((\mathbf{q}_1+\mathbf{q}_2+\mathbf{q}_3).\mathbf{r}_l\right)\right)\end{bmatrix}+CC\right)$$

$$+\sum_{j,j',j'',\alpha,\beta,\gamma}\sum_{p_1,p_2,p_3}\sum_{\mathbf{q}_1,\mathbf{q}_2,\mathbf{q}_3}\left(\begin{bmatrix}\sum_{h'h''}\frac{G_{j'',h'',\gamma,j',h',\beta,j,l,\alpha}}{3!}\exp\left(i\left(\mathbf{q}_2.\mathbf{r}_{h'}\right)\right)\exp\left(i\left(-\mathbf{q}_3.\mathbf{r}_{h''}\right)\right)\\ e_{j,\alpha}(\mathbf{q}_1,p_1)e_{j',\beta}(\mathbf{q}_2,p_2)e^*_{j'',\gamma}(\mathbf{q}_3,p_3)\\ A_+(\mathbf{q}_1,p_1,t)A_+(\mathbf{q}_2,p_2,t)A^*_+(\mathbf{q}_3,p_3,t)\\ \exp\left(-i\left(\left[w(\mathbf{q}_1,p_1)+w(\mathbf{q}_2,p_2)-w(\mathbf{q}_3,p_3)\right]t\right)\right)\\ \sum_l\exp\left(i\left((\mathbf{q}_1+\mathbf{q}_2-\mathbf{q}_3).\mathbf{r}_l\right)\right)\end{bmatrix}+CC\right)$$

$$+\sum_{j,j',j'',\alpha,\beta,\gamma}\sum_{p_1,p_2,p_3}\sum_{\mathbf{q}_1,\mathbf{q}_2,\mathbf{q}_3}\left(\begin{bmatrix}\sum_{h'h''}\frac{G_{j'',h'',\gamma,j',h',\beta,j,l,\alpha}}{3!}\exp\left(i\left(-\mathbf{q}_2.\mathbf{r}_{h'}\right)\right)\exp\left(i\left(\mathbf{q}_3.\mathbf{r}_{h''}\right)\right)\\ e_{j,\alpha}(\mathbf{q}_1,p_1)e^*_{j',\beta}(\mathbf{q}_2,p_2)e_{j'',\gamma}(\mathbf{q}_3,p_3)\\ A_+(\mathbf{q}_1,p_1,t)A^*_+(\mathbf{q}_2,p_2,t)A_+(\mathbf{q}_3,p_3,t)\\ \exp\left(-i\left(\left[w(\mathbf{q}_1,p_1)-w(\mathbf{q}_2,p_2)+w(\mathbf{q}_3,p_3)\right]t\right)\right)\\ \sum_l\exp\left(i\left((\mathbf{q}_1-\mathbf{q}_2+\mathbf{q}_3).\mathbf{r}_l\right)\right)\end{bmatrix}+CC\right)$$

$$+\sum_{j,j',j'',\alpha,\beta,\gamma}\sum_{p_1,p_2,p_3}\sum_{\mathbf{q}_1,\mathbf{q}_2,\mathbf{q}_3}\left(\begin{bmatrix}\sum_{h'h''}\frac{G_{j'',h'',\gamma,j',h',\beta,j,l,\alpha}}{3!}\exp\left(i\left(-\mathbf{q}_2.\mathbf{r}_{h'}\right)\right)\exp\left(i\left(-\mathbf{q}_3.\mathbf{r}_{h''}\right)\right)\\ e_{j,\alpha}(\mathbf{q}_1,p_1)e^*_{j',\beta}(\mathbf{q}_2,p_2)e^*_{j'',\gamma}(\mathbf{q}_3,p_3)\\ A_+(\mathbf{q}_1,p_1,t)A^*_+(\mathbf{q}_2,p_2,t)A^*_+(\mathbf{q}_3,p_3,t)\\ \exp\left(-i\left(\left[w(\mathbf{q}_1,p_1)-w(\mathbf{q}_2,p_2)-w(\mathbf{q}_3,p_3)\right]t\right)\right)\\ \sum_l\exp\left(i\left((\mathbf{q}_1-\mathbf{q}_2-\mathbf{q}_3).\mathbf{r}_l\right)\right)\end{bmatrix}+CC\right) \tag{S10}$$

The summation over h', h'' is constant, Therefore:

$$F^{j,j',j''}_{\alpha,\beta,\gamma}(\mathbf{q}_2,\mathbf{q}_3)=\sum_{h'h''}\frac{G_{j'',h'',\gamma,j',h',\beta,j,l,\alpha}}{3!}\exp\left(i\left(\mathbf{q}_2.\mathbf{r}_{h'}\right)\right)\exp\left(i\left(\mathbf{q}_3.\mathbf{r}_{h''}\right)\right) \tag{S11}$$

Substituting Eqn. (S11) in (S10) and simplifying:

$$H_3(t) = \sum_{j,j',j'',\alpha,\beta,\gamma} \sum_{p_1,p_2,p_3} \sum_{\mathbf{q}_1,\mathbf{q}_2,\mathbf{q}_3} \left(\begin{bmatrix} F^{j,j',j''}_{\alpha,\beta,\gamma}(\mathbf{q}_2,\mathbf{q}_3) e_{j,\alpha}(\mathbf{q}_1,p_1) e_{j',\beta}(\mathbf{q}_2,p_2) e_{j'',\gamma}(\mathbf{q}_3,p_3) \\ A_+(\mathbf{q}_1,p_1,t) A_+(\mathbf{q}_2,p_2,t) A_+(\mathbf{q}_3,p_3,t) \\ \exp(-i([w(\mathbf{q}_1,p_1) + w(\mathbf{q}_2,p_2) + w(\mathbf{q}_3,p_3)]t)) \\ \sum_l \exp(i((\mathbf{q}_1 + \mathbf{q}_2 + \mathbf{q}_3).\mathbf{r}_l)) \end{bmatrix} + CC \right)$$
$$+ \sum_{j,j',j'',\alpha,\beta,\gamma} \sum_{p_1,p_2,p_3} \sum_{\mathbf{q}_1,\mathbf{q}_2,\mathbf{q}_3} \left(\begin{bmatrix} F^{j,j',j''}_{\alpha,\beta,\gamma}(\mathbf{q}_2,-\mathbf{q}_3) e_{j,\alpha}(\mathbf{q}_1,p_1) e_{j',\beta}(\mathbf{q}_2,p_2) e^*_{j'',\gamma}(\mathbf{q}_3,p_3) \\ A_+(\mathbf{q}_1,p_1,t) A_+(\mathbf{q}_2,p_2,t) A^*_+(\mathbf{q}_3,p_3,t) \\ \exp(-i([w(\mathbf{q}_1,p_1) + w(\mathbf{q}_2,p_2) - w(\mathbf{q}_3,p_3)]t)) \\ \sum_l \exp(i((\mathbf{q}_1 + \mathbf{q}_2 - \mathbf{q}_3).\mathbf{r}_l)) \end{bmatrix} + CC \right)$$
$$+ \sum_{j,j',j'',\alpha,\beta,\gamma} \sum_{p_1,p_2,p_3} \sum_{\mathbf{q}_1,\mathbf{q}_2,\mathbf{q}_3} \left(\begin{bmatrix} F^{j,j',j''}_{\alpha,\beta,\gamma}(-\mathbf{q}_2,\mathbf{q}_3) e_{j,\alpha}(\mathbf{q}_1,p_1) e^*_{j',\beta}(\mathbf{q}_2,p_2) e_{j'',\gamma}(\mathbf{q}_3,p_3) \\ A_+(\mathbf{q}_1,p_1,t) A^*_+(\mathbf{q}_2,p_2,t) A_+(\mathbf{q}_3,p_3,t) \\ \exp(-i([w(\mathbf{q}_1,p_1) - w(\mathbf{q}_2,p_2) + w(\mathbf{q}_3,p_3)]t)) \\ \sum_l \exp(i((\mathbf{q}_1 - \mathbf{q}_2 + \mathbf{q}_3).\mathbf{r}_l)) \end{bmatrix} + CC \right)$$
$$+ \sum_{j,j',j'',\alpha,\beta,\gamma} \sum_{p_1,p_2,p_3} \sum_{\mathbf{q}_1,\mathbf{q}_2,\mathbf{q}_3} \left(\begin{bmatrix} F^{j,j',j''}_{\alpha,\beta,\gamma}(-\mathbf{q}_2,-\mathbf{q}_3) e_{j,\alpha}(\mathbf{q}_1,p_1) e^*_{j',\beta}(\mathbf{q}_2,p_2) e^*_{j'',\gamma}(\mathbf{q}_3,p_3) \\ A_+(\mathbf{q}_1,p_1,t) A^*_+(\mathbf{q}_2,p_2,t) A^*_+(\mathbf{q}_3,p_3,t) \\ \exp(-i([w(\mathbf{q}_1,p_1) - w(\mathbf{q}_2,p_2) - w(\mathbf{q}_3,p_3)]t)) \\ \sum_l \exp(i((\mathbf{q}_1 - \mathbf{q}_2 - \mathbf{q}_3).\mathbf{r}_l)) \end{bmatrix} + CC \right) \quad \text{(S12)}$$

The summation over l goes to zero unless the corresponding wave vectors add up to a reciprocal lattice vector. Therefore:

$$H_3(t) = \sum_{j,j',j'',\alpha,\beta,\gamma} \sum_{p_1,p_2,p_3} \sum_{\mathbf{q}_1+\mathbf{q}_2+\mathbf{q}_3=\mathbf{g}} \left(\begin{bmatrix} F^{j,j',j''}_{\alpha,\beta,\gamma}(\mathbf{q}_2,\mathbf{q}_3) e_{j,\alpha}(\mathbf{q}_1,p_1) e_{j',\beta}(\mathbf{q}_2,p_2) e_{j'',\gamma}(\mathbf{q}_3,p_3) \\ A_+(\mathbf{q}_1,p_1,t) A_+(\mathbf{q}_2,p_2,t) A_+(\mathbf{q}_3,p_3,t) \\ \exp(-i([w(\mathbf{q}_1,p_1) + w(\mathbf{q}_2,p_2) + w(\mathbf{q}_3,p_3)]t)) \end{bmatrix} + CC \right)$$
$$+ \sum_{j,j',j'',\alpha,\beta,\gamma} \sum_{p_1,p_2,p_3} \sum_{\mathbf{q}_1+\mathbf{q}_2-\mathbf{q}_3=\mathbf{g}} \left(\begin{bmatrix} F^{j,j',j''}_{\alpha,\beta,\gamma}(\mathbf{q}_2,-\mathbf{q}_3) e_{j,\alpha}(\mathbf{q}_1,p_1) e_{j',\beta}(\mathbf{q}_2,p_2) e^*_{j'',\gamma}(\mathbf{q}_3,p_3) \\ A_+(\mathbf{q}_1,p_1,t) A_+(\mathbf{q}_2,p_2,t) A^*_+(\mathbf{q}_3,p_3,t) \\ \exp(-i([w(\mathbf{q}_1,p_1) + w(\mathbf{q}_2,p_2) - w(\mathbf{q}_3,p_3)]t)) \end{bmatrix} + CC \right)$$
$$+ \sum_{j,j',j'',\alpha,\beta,\gamma} \sum_{p_1,p_2,p_3} \sum_{\mathbf{q}_1-\mathbf{q}_2+\mathbf{q}_3=\mathbf{g}} \left(\begin{bmatrix} F^{j,j',j''}_{\alpha,\beta,\gamma}(-\mathbf{q}_2,\mathbf{q}_3) e_{j,\alpha}(\mathbf{q}_1,p_1) e^*_{j',\beta}(\mathbf{q}_2,p_2) e_{j'',\gamma}(\mathbf{q}_3,p_3) \\ A_+(\mathbf{q}_1,p_1,t) A^*_+(\mathbf{q}_2,p_2,t) A_+(\mathbf{q}_3,p_3,t) \\ \exp(-i([w(\mathbf{q}_1,p_1) - w(\mathbf{q}_2,p_2) + w(\mathbf{q}_3,p_3)]t)) \end{bmatrix} + CC \right)$$
$$+ \sum_{j,j',j'',\alpha,\beta,\gamma} \sum_{p_1,p_2,p_3} \sum_{\mathbf{q}_1-\mathbf{q}_2-\mathbf{q}_3=\mathbf{g}} \left(\begin{bmatrix} F^{j,j',j''}_{\alpha,\beta,\gamma}(-\mathbf{q}_2,-\mathbf{q}_3) e_{j,\alpha}(\mathbf{q}_1,p_1) e^*_{j',\beta}(\mathbf{q}_2,p_2) e^*_{j'',\gamma}(\mathbf{q}_3,p_3) \\ A_+(\mathbf{q}_1,p_1,t) A^*_+(\mathbf{q}_2,p_2,t) A^*_+(\mathbf{q}_3,p_3,t) \\ \exp(-i([w(\mathbf{q}_1,p_1) - w(\mathbf{q}_2,p_2) - w(\mathbf{q}_3,p_3)]t)) \end{bmatrix} + CC \right) \quad \text{(S13)}$$

Note that the extra factor of N_u has been merged into F. Rearranging the indices and simplifying:

$$H_3(t)=\sum_{p_1,p_2,p_3}\sum_{\mathbf{q}_1+\mathbf{q}_2+\mathbf{q}_3=\mathbf{g}}\sum_{j,j',j'',\alpha,\beta,\gamma}F_{\alpha,\beta,\gamma}^{j,j',j''}(\mathbf{q}_2,\mathbf{q}_3)e_{j,\alpha}(\mathbf{q}_1,p_1)e_{j',\beta}(\mathbf{q}_2,p_2)e_{j'',\gamma}(\mathbf{q}_3,p_3)\times\left[\begin{array}{l}\left(\begin{array}{l}A_+(\mathbf{q}_1,p_1,t)A_+(\mathbf{q}_2,p_2,t)A_+(\mathbf{q}_3,p_3,t)\\ \exp\left(-i\left(\left[w(\mathbf{q}_1,p_1)+w(\mathbf{q}_2,p_2)+w(\mathbf{q}_3,p_3)\right]t\right)\right)\end{array}\right)\\ +\left(\begin{array}{l}A_+(\mathbf{q}_1,p_1,t)A_+(\mathbf{q}_2,p_2,t)A_+^*(-\mathbf{q}_3,p_3,t)\\ \exp\left(-i\left(\left[w(\mathbf{q}_1,p_1)+w(\mathbf{q}_2,p_2)-w(\mathbf{q}_3,p_3)\right]t\right)\right)\end{array}\right)\\ +\left(\begin{array}{l}A_+(\mathbf{q}_1,p_1,t)A_+^*(-\mathbf{q}_2,p_2,t)A_+(\mathbf{q}_3,p_3,t)\\ \exp\left(-i\left(\left[w(\mathbf{q}_1,p_1)-w(\mathbf{q}_2,p_2)+w(\mathbf{q}_3,p_3)\right]t\right)\right)\end{array}\right)\\ +\left(\begin{array}{l}A_+(\mathbf{q}_1,p_1,t)A_+^*(-\mathbf{q}_2,p_2,t)A_+^*(-\mathbf{q}_3,p_3,t)\\ \exp\left(-i\left(\left[w(\mathbf{q}_1,p_1)-w(\mathbf{q}_2,p_2)-w(\mathbf{q}_3,p_3)\right]t\right)\right)\end{array}\right)\end{array}\right]+CC \qquad \text{(S14)}$$

Separating out the amplitudes and phase terms:

$$H_3(t)=\sum_{p_1,p_2,p_3}\sum_{\mathbf{q}_1+\mathbf{q}_2+\mathbf{q}_3=\mathbf{g}}\left|L\left(\begin{smallmatrix}p_1,p_2,p_3\\ \mathbf{q}_1,\mathbf{q}_2,\mathbf{q}_3\end{smallmatrix}\right)\right|\exp\left(i\phi_L\left(\begin{smallmatrix}p_1,p_2,p_3\\ \mathbf{q}_1,\mathbf{q}_2,\mathbf{q}_3\end{smallmatrix}\right)\right)\times\left[\begin{array}{l}\left(\begin{array}{l}|A_+(\mathbf{q}_1,p_1,t)||A_+(\mathbf{q}_2,p_2,t)||A_+(\mathbf{q}_3,p_3,t)|\\ \exp\left(i\left(\phi_+(\mathbf{q}_1,p_1)+\phi_+(\mathbf{q}_2,p_2)+\phi_+(\mathbf{q}_3,p_3)\right)\right)\\ \exp\left(-i\left(\left[w(\mathbf{q}_1,p_1)+w(\mathbf{q}_2,p_2)+w(\mathbf{q}_3,p_3)\right]t\right)\right)\end{array}\right)\\ +\left(\begin{array}{l}|A_+(\mathbf{q}_1,p_1,t)||A_+(\mathbf{q}_2,p_2,t)||A_+(-\mathbf{q}_3,p_3,t)|\\ \exp\left(i\left(\phi_+(\mathbf{q}_1,p_1)+\phi_+(\mathbf{q}_2,p_2)-\phi_+(-\mathbf{q}_3,p_3)\right)\right)\\ \exp\left(-i\left(\left[w(\mathbf{q}_1,p_1)+w(\mathbf{q}_2,p_2)-w(\mathbf{q}_3,p_3)\right]t\right)\right)\end{array}\right)\\ +\left(\begin{array}{l}|A_+(\mathbf{q}_1,p_1,t)||A_+(-\mathbf{q}_2,p_2,t)||A_+(\mathbf{q}_3,p_3,t)|\\ \exp\left(i\left(\phi_+(\mathbf{q}_1,p_1)-\phi_+(-\mathbf{q}_2,p_2)+\phi_+(\mathbf{q}_3,p_3)\right)\right)\\ \exp\left(-i\left(\left[w(\mathbf{q}_1,p_1)-w(\mathbf{q}_2,p_2)+w(\mathbf{q}_3,p_3)\right]t\right)\right)\end{array}\right)\\ +\left(\begin{array}{l}|A_+(\mathbf{q}_1,p_1,t)||A_+(-\mathbf{q}_2,p_2,t)||A_+(-\mathbf{q}_3,p_3,t)|\\ \exp\left(i\left(\phi_+(\mathbf{q}_1,p_1)-\phi_+(-\mathbf{q}_2,p_2)-\phi_+(-\mathbf{q}_3,p_3)\right)\right)\\ \exp\left(-i\left(\left[w(\mathbf{q}_1,p_1)-w(\mathbf{q}_2,p_2)-w(\mathbf{q}_3,p_3)\right]t\right)\right)\end{array}\right)\end{array}\right]+CC \qquad \text{(S15)}$$

where

$$A_+(\mathbf{q}_1,p_1,t)=|A_+(\mathbf{q}_1,p_1,t)|\exp\left(i\left(\phi_+(\mathbf{q}_1,p_1)\right)\right) \qquad \text{(S16)}$$

$$L\left(\begin{smallmatrix}p_1,p_2,p_3\\ \mathbf{q}_1,\mathbf{q}_2,\mathbf{q}_3\end{smallmatrix}\right)=\sum_{j,j',j'',\alpha,\beta,\gamma}F_{\alpha,\beta,\gamma}^{j,j',j''}(\mathbf{q}_2,\mathbf{q}_3)e_{j,\alpha}(\mathbf{q}_1,p_1)e_{j',\beta}(\mathbf{q}_2,p_2)e_{j'',\gamma}(\mathbf{q}_3,p_3)=\left|L\left(\begin{smallmatrix}p_1,p_2,p_3\\ \mathbf{q}_1,\mathbf{q}_2,\mathbf{q}_3\end{smallmatrix}\right)\right|\exp\left(i\phi_L\left(\begin{smallmatrix}p_1,p_2,p_3\\ \mathbf{q}_1,\mathbf{q}_2,\mathbf{q}_3\end{smallmatrix}\right)\right) \qquad \text{(S17)}$$

Merging the phases and adding the complex conjugate (CC), and rewriting in terms of real mode amplitudes $A(\mathbf{q},p,t)$ from the cosine solution in Eqn. (4) of the main manuscript:

$$A(\mathbf{q},p,t)=\left.\begin{matrix} 2\left|A_{+}(\mathbf{q},p,t)\right| \forall \phi_{+}(\mathbf{q},p)\in[0,\pi) \\ -2\left|A_{+}(\mathbf{q},p,t)\right| \forall \phi_{+}(\mathbf{q},p)\in[\pi,2\pi) \end{matrix}\right\} \tag{S18}$$

$$H_3(t)=\sum_{p_1,p_2,p_3}\sum_{\mathbf{q}_1+\mathbf{q}_2+\mathbf{q}_3=\mathbf{g}}\left[\begin{matrix} \left(\begin{matrix}\left|L\left(\begin{smallmatrix}p_1,p_2,p_3\\ \mathbf{q}_1,\mathbf{q}_2,\mathbf{q}_3\end{smallmatrix}\right)\right| A(\mathbf{q}_1,p_1,t)A(\mathbf{q}_2,p_2,t)A(\mathbf{q}_3,p_3,t) \\ \cos\left(\left(w(\mathbf{q}_1,p_1)+w(\mathbf{q}_2,p_2)+w(\mathbf{q}_3,p_3)\right)t+\phi\left(\begin{smallmatrix}p_1,p_2,p_3\\ \mathbf{q}_1,\mathbf{q}_2,\mathbf{q}_3\end{smallmatrix}\right)\right)\end{matrix}\right) \\ +\left(\begin{matrix}\left|L\left(\begin{smallmatrix}p_1,p_2,p_3\\ \mathbf{q}_1,\mathbf{q}_2,\mathbf{q}_3\end{smallmatrix}\right)\right| A(\mathbf{q}_1,p_1,t)A(\mathbf{q}_2,p_2,t)A(-\mathbf{q}_3,p_3,t) \\ \cos\left(\left(w(\mathbf{q}_1,p_1)+w(\mathbf{q}_2,p_2)-w(\mathbf{q}_3,p_3)\right)t+\phi\left(\begin{smallmatrix}p_1,p_2,p_3\\ \mathbf{q}_1,\mathbf{q}_2,-\mathbf{q}_3\end{smallmatrix}\right)\right)\end{matrix}\right) \\ +\left(\begin{matrix}\left|L\left(\begin{smallmatrix}p_1,p_2,p_3\\ \mathbf{q}_1,\mathbf{q}_2,\mathbf{q}_3\end{smallmatrix}\right)\right| A(\mathbf{q}_1,p_1,t)A(-\mathbf{q}_2,p_2,t)A(\mathbf{q}_3,p_3,t) \\ \cos\left(\left(w(\mathbf{q}_1,p_1)-w(\mathbf{q}_2,p_2)+w(\mathbf{q}_3,p_3)\right)t+\phi\left(\begin{smallmatrix}p_1,p_2,p_3\\ \mathbf{q}_1,-\mathbf{q}_2,\mathbf{q}_3\end{smallmatrix}\right)\right)\end{matrix}\right) \\ +\left(\begin{matrix}\left|L\left(\begin{smallmatrix}p_1,p_2,p_3\\ \mathbf{q}_1,\mathbf{q}_2,\mathbf{q}_3\end{smallmatrix}\right)\right| A(\mathbf{q}_1,p_1,t)A(-\mathbf{q}_2,p_2,t)A(-\mathbf{q}_3,p_3,t) \\ \cos\left(\left(w(\mathbf{q}_1,p_1)-w(\mathbf{q}_2,p_2)-w(\mathbf{q}_3,p_3)\right)t+\phi\left(\begin{smallmatrix}p_1,p_2,p_3\\ \mathbf{q}_1,-\mathbf{q}_2,-\mathbf{q}_3\end{smallmatrix}\right)\right)\end{matrix}\right)\end{matrix}\right] \tag{S19}$$

The cubic Hamiltonian can now be more compactly expressed as:

$$H_3(t)=\sum_{\mathbf{q},p}\left[\begin{matrix}\left(L\times A(\mathbf{q}_1,t)A(\mathbf{q}_2,t)A(\mathbf{q}_3,t)\cos\left(\left(w(\mathbf{q}_1)+w(\mathbf{q}_2)+w(\mathbf{q}_3)\right)t+\phi_1\right)\right)+ \\ \left(L\times A(\mathbf{q}_1,t)A(\mathbf{q}_2,t)A(-\mathbf{q}_3,t)\cos\left(\left(w(\mathbf{q}_1)+w(\mathbf{q}_2)-w(\mathbf{q}_3)\right)t+\phi_2\right)\right)+ \\ \left(L\times A(\mathbf{q}_1,t)A(-\mathbf{q}_2,t)A(\mathbf{q}_3,t)\cos\left(\left(w(\mathbf{q}_1)-w(\mathbf{q}_2)+w(\mathbf{q}_3)\right)t+\phi_3\right)\right)+ \\ \left(L\times A(\mathbf{q}_1,t)A(-\mathbf{q}_2,t)A(-\mathbf{q}_3,t)\cos\left(\left(w(\mathbf{q}_1)-w(\mathbf{q}_2)-w(\mathbf{q}_3)\right)t+\phi_4\right)\right)\end{matrix}\right] \tag{S20}$$

Eqn. (S20), which is given by Eqn. (1) of the main manuscript, highlights the frequency relationships that become apparent with the asymmetric real normal mode amplitudes.

References

1 Tersoff, J. Empirical interatomic potential for carbon, with applications to amorphous carbon. *Phys. Rev. Lett.* **61**, 2879 (1988).

2 Lindsay, L. & Broido, D. A. Optimized Tersoff and Brenner empirical potential parameters for lattice dynamics and phonon thermal transport in carbon nanotubes and graphene. *Phys. Rev. B* **81**, 205441 (2010).

3 Raj, A. & Eapen, J. Phonon dispersion using the ratio of zero-time correlations among conjugate variables: Computing full phonon dispersion surface of graphene. *Comput. Phys. Commun* **238**, 124 (2019).

4 Srivastava, G. P. *The physics of phonons*. (Taylor and Francis Group, 1990).
